# A whole transcriptome profiling analysis for antidepressant mechanism of Xiaoyaosan mediated synapse loss via BDNF/trkB/PI3K signal axis in CUMS rats

**DOI:** 10.1186/s12906-023-04000-0

**Published:** 2023-06-15

**Authors:** Pan Meng, Xi Zhang, Tong-tong Liu, Jian Liu, Yan Luo, Ming-xia Xie, Hui Yang, Rui Fang, Dong-wei Guo, Zi-yan Zhong, Yu-hong Wang, Jin-Wen Ge

**Affiliations:** 1 Hunan University of Chinese Medicine, 300 Xueshi Road, Hanpu Science and Education Park, Yuelu District, Hunan Changsha, China; 2 The Second People’s Hospital of Hunan Province, Changsha, Hunan China; 3 First Affiliated Hospital, Hunan University of Chinese Medicine, Changsha, Hunan China; 4 Hunan Academy of Chinese Medicine, Yuelu District, 58 Lushan Road, Changsha, Hunan China

**Keywords:** Xiaoyaosan, Depression, Whole transcriptomic analysis, Synapse loss, BDNF/trkB/PI3K signal axis

## Abstract

**Background:**

Depression is a neuropsychiatric disease resulting from deteriorations of molecular networks and synaptic injury induced by stress. Traditional Chinese formula Xiaoyaosan (XYS) exert antidepressant effect, which was demonstrated by a great many of clinical and basic investigation. However, the exact mechanism of XYS has not yet been fully elucidated.

**Methods:**

In this study, chronic unpredictable mild stress (CUMS) rats were used as a model of depression. Behavioral test and HE staining were used to detect the anti-depressant effects of XYS. Furthermore, whole transcriptome sequencing was employed to establish the microRNA (miRNA), long non-coding RNA (lncRNA), circular RNA (circRNA), and mRNA profiles. The biological functions and potential mechanisms of XYS for depression were gathered from the GO and KEGG pathway. Then, constructed the competing endogenous RNA (ceRNA) networks to illustrate the regulatory relationship between non-coding RNA (ncRNA) and mRNA. Additionally, longest dendrite length, total length of dendrites, number of intersections, and density of dendritic spines were detected by Golgi staining. MAP2, PSD-95, SYN were detected by immunofluorescence respectively. BDNF, TrkB, p-TrkB, PI3K, Akt, p-Akt were measured by Western Blotting.

**Results:**

The results showed that XYS could increase the locomotor activity and sugar preference, decreased swimming immobility time as well as attenuate hippocampal pathological damage. A total of 753 differentially expressed lncRNAs (DElncRNAs), 28 circRNAs (DEcircRNAs), 101 miRNAs (DEmiRNAs), and 477 mRNAs (DEmRNAs) were identified after the treatment of XYS in whole transcriptome sequencing analysis. Enrichment results revealed that XYS could regulate multiple aspects of depression through different synapse or synaptic associated signal, such as neurotrophin signaling and PI3K/Akt signaling pathways. Then, vivo experiments indicated that XYS could promote length, density, intersections of synapses and also increase the expression of MAP2 in hippocampal CA1, CA3 regions. Meanwhile, XYS could increase the expression of PSD-95, SYN in the CA1, CA3 regions of hippocampal by regulating the BDNF/trkB/PI3K signal axis.

**Conclusion:**

The possible mechanism on synapse of XYS in depression was successfully predicted. BDNF/trkB/PI3K signal axis were the potential mechanism of XYS on synapse loss for its antidepressant. Collectively, our results provided novel information about the molecular basis of XYS in treating depression.

**Supplementary Information:**

The online version contains supplementary material available at 10.1186/s12906-023-04000-0.

## Background

Depression is a psychological illness worldwide which characterized by a depressed mood and cognitive dysfunction. According to the World Health Organization (WHO) report, depression will become the main cause of disability by the end of 2030 [1]. In addition, depression which is a chronic and life-threatening mental disorder affects almost 350 million people in the world and leads to a substantial economic burden on society and families. However, there are some limitations in current antidepressants, such as low response rate and some side effects [2].

Xiaoyaosan (XYS), a classic formula in traditional Chinese medicine (TCM), has been used for the treatment of depression for more than 2000 years from the Song dynasty in China. According to the clinical and basic science studies, XYS exerts good antidepressant effect such as decrease the Hamilton Depression Rating Scale (HAMD) in patients [3], ameliorate chronic unpredictable mild stress (CUMS)-induced depression-like behaviors in mice or rats [4, 5]. Many studies have shown that XYS exerts antidepressant by regulating the functions of hippocampus, such as astrocytes, microglia, necroptosis and neurogenesis [6–8]. Meanwhile, it has been demonstrated that XYS can alleviates glutamate-mediated toxicity in CUMS through NR2B and PI3K/Akt signaling pathways [9], also XYS could ameliorate hippocampal neuron damage through acting on Cx43/GR/BDNF axis [10]. However, the exact mechanism of XYS has not been fully clarified.

Non-coding RNAs (ncRNAs) belong to the RNA family which do not encode proteins typically. ncRNAs including microRNAs (miRNAs), lncRNAs and circRNAs, which play key roles in the development and progression of depression [11], and adjust the information transmission from genotype to phenotypic status [12]. Gaining insight into these ncRNAs will contribute not only to promoting treatment and drug development but also to understanding the biological mechanisms of depression. Previous studies have shown that ncRNAs, especially lncRNAs and miRNAs, can affect the occurrence and development of depression by regulating brain development, homeostasis, stress response and synaptic plasticity [13]. In addition, miRNA and circRNA can restore their abnormal functions in depression by regulating specific signals, which may imply the impairment of genes implicated in pathways of MDD etiopathogenesis, such as neuroinflammation, brain-derived neurotrophic factor (BDNF), neurotransmitters, hypothalamic–pituitary–adrenal (HPA) axis, PI3K/Akt signal, and et al. [14]. However, few of transcriptomes concerned about the transcriptomic effect of XYS intervention against CUMS were published, and various questions remain unanswered with respect to the ncRNAs in response to XYS treatment.

In this study, the whole transcriptome sequencing technique was used to profile the coding transcriptome and ncRNAs changes occurred in CUMS rats response to XYS treatment, and to explore the biological roles and potential signaling pathways. Then, the animal experiments was observed to verify the antidepressant mechanism through CUMS rats.

## Materials and methods

### Animals and grouping

**Fig. 1:**
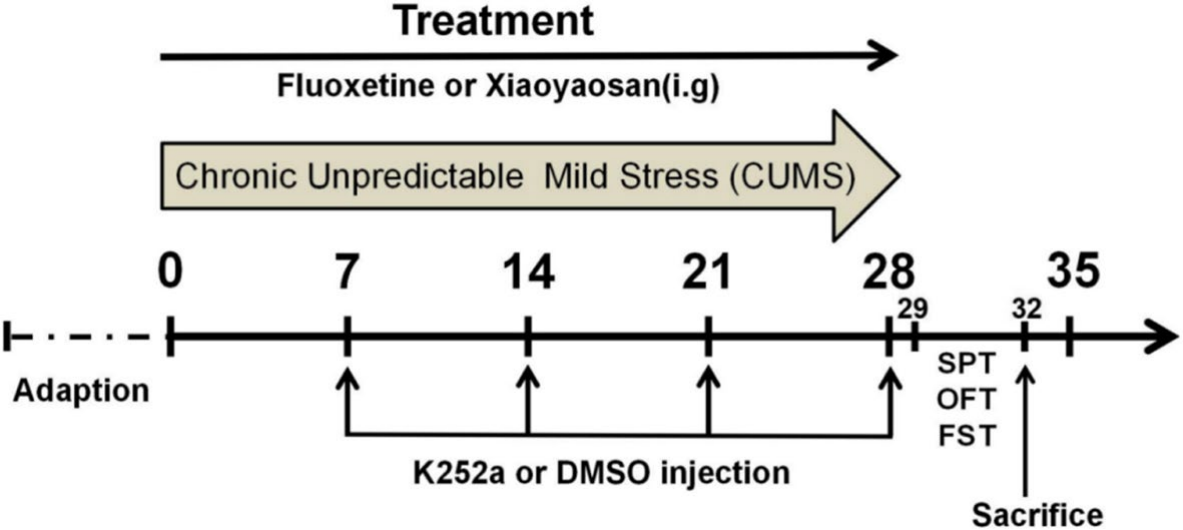
A schematic diagram showing the experimental design. After 1 week of habituation, rats were subjected to CUMS procedure for four weeks. Except for the control group, the rats in the other groups were subjected to CUMS for 4 weeks and treated with different drugs. Fluoxetine (5.4 mg/kg/day) or Xiaoyaosan (2.224 g/kg/day) were administered i.g once a day. The K252a or DMSO were intrahippocampal microinjected on the 7th, 14th, 21th, 28th day respectively, then OFT, SPT, FST were implemented. Then, the rats were sacrificed for further detection

Male Sprague–Dawley rats, approximately 5 weeks old and weighting 180–200 g were purchased from the Hunan Slake Jingda experimental animal Co., Ltd (changsha, China). The protocol was approved by the Institutional Animal Care and Use Committee of Hunan University of Chinese Medicine, and in accordance with the guidelines for animal welfare (HNCM-LL-2020111001). The rats were housed in an animal room with a barrier system, and free access to water and food for 7 days before the experiments. The rats were randomly divided into six groups: including control group, CUMS group, fluoxetine group (cat#: 0918A, Tasly Pharmaceutical Co., Ltd. Tianjin, China), XYS group (cat#: J2194, Jiuzhitang Co., Ltd,Changsha, China), vehicle + CUMS group, XYS + K252a group with 10 rats in each group. The rats in the XYS + K252a groups received an intrahippocampal microinjection of K252a (5 mM dissolved in DMSO, cat#: K1639, sigma) at a volume of 5μL at a speed of 1 μL/min [15], K252a inject once every week with a total of four times. The rats in vehicle group received CUMS as well as an intrahippocampal microinjection of DMSO, the injection time and volume are consistent with K252a. All groups were subjected to a variety of stressors for four consecutive weeks except the control group, and the control group had free access to water and food. The experimental procedure is shown in Fig. 1.

The CUMS procedure stressors referred to our previous research [16]. Rats were individually housed and underwent a variable sequence of unpredictable and mild stimulation for 4 weeks, which included 24 h food deprivation, 24 h water deprivation, 1 min tail pinching, 12-h cage tilt at 45°from the horizontal, electric shock to the foot (50 V, administered every other minute and lasting 10 s), swimming at 4℃ in ice water for 4 min, 12-h noise(85 dB), 24 h reversed light/dark cycle. The rats received one of the stressors every day (8:00–12:00 am), and the same type of stressor did not occur on three consecutive days to prevent the animals from anticipating the occurrence of the stimuli.

### Preparation and determination of XYS

XYS is formulated by eight traditional Chinese medicinal herbs, i.e., *Radix Bupleuri* (Chaihu in TCM) 9 g*, Radix Angelicae sinensis* (Danggui in TCM) 9 g*, Radix Paeoniae alba* (Baishao in TCM) 9 g*, Rhizome Atractylodis macrocephala* (ChaoBaizhu in TCM) 9 g, *Poria cocos* (Fuling in TCM) 9 g, *Herba Menthae haplocalycis* (Bohe in TCM) 3 g, *Rhizome Zingiberis officinale* (Shengjiang in TCM) 6 g, and *Radix Glycyrrhizae preparata* (ZhiGancao in TCM) 4.5 g*.* XYS dry powder was prepared by Jiuzhitang Co., Ltd,Changsha, China (Lot No. J2194), in accord with the process recorded in the Chinese Pharmacopoeia 2015 Edition. 1.0 g of crude drug yielded 0.476 g the finished dry powder administered experimentally.

To assure the quality and stability of XYS, LC–MS/MS method was performed to detected the chemical composition and simultaneous determination of four active consitituents (Albiflorin, Paeoniflorin, Ligustilide, Glycyrrhizic acid). The 1.0 g of XYS was dissolved in 20 mL methanol, and filtered via 0.22 μm filter cartridge. Accurately weighed standard compounds were dissolved in methanol to a specific concentration. The mobile phase was made up of A (0.1% formic acid in water, v/v) and B (acetonitrile). Gradient elution was as follows: 0–5 min (5–15% B), 5–10 min (15–25% B), 10–20 min (25–45% B), 20–30 min (45–65% B), 30–40 min (65–95% B) with the flow rate at 0.4 mL/min and the temperature of column was 25 °C. The parameter of ESI negative and positive-ion mode was ion source gas 1 and ion source gas 210 psi, curtain gas 35 psi. Finally, the quantitative analysis was employed by Agilent 1290 UPLC-6540-Q-TOF.

### Behavioral testing

#### Open field test (OFT)

An open field box (100 cm × 100 cm × 50 cm) with a black floor divided into 25 smaller areas and four black sidewalls. Rats individually were placed into the center of box and allow them to explore freely. After 30 s of acclimation, record the changes of horizontal activities, vertical activities, grooming number, rest time and defecating number within 5 min. The horizontal activities as indicated by transition of all four feet into a new area, and the number of the front limb leaving the ground is vertical activity.

#### Forced swim test (FST)

Rats were placed in a transparent cylindrical acrylic container (60 cm high and 20 cm in diameter), which was filled with tap water (22–24℃, 45 cm high) for 5 min swimming acclimation. The immobility time of the hind limbs were counted in the last 4 min. Accumulating immobility time was used to assess despair and desperate behavior of rats.

#### Sucrose preference test (SPT)

Rats were presented with two bottles at the same time, one containing water, the other containing sucrose solution (1%, w/v), and acclimated to the two bottles test choice in 24 h before testing. All rats drank both the water and the 1% sucrose solution, then deprived of water and food for 23 h. Sucrose preference test was conducted in which rats were housed in individual cages and were free to access to two bottles containing 100 ml of sucrose solution (1%, w/v) and 100 ml of water. After 1 h, the volumes of consumed sucrose solution and water were recorded and the sucrose preference was calculated as the sucrose preference (%) = sucrose consumption / (sucrose consumption + water consumption).

### HE staining

Brain tissues were fixed with 4% paraformaldehyde overnight, paraffin embedded and sliced into sections of 5 μm. The sections were dewaxed to water, rehydrated with graded concentrations of ethanol and then stained with haematoxy‐lin and eosin. An inverted microscope (Zeiss, Axio Scope A1) was used for observation.

### Whole transcriptome sequencing and profiling

The rats were sacrificed, the hippocampal tissue was stripped on ice, then quickly frozen into liquid nitrogen and transferred to -80℃ refrigerator. RNA was extracted from hippocampus and quality monitoring. Construct a small RNA Library (sRNA library) and reverse transcribe RNA to synthesize cDNA, then followed by sequencing on the Illumina HiSeq platform after the quality tests. Based on Sequencing-By-Synthesis (SBS) technology, the Illumina HiSeq high-throughput sequencing platform sequenced cDNA libraries to produce a large amount of high-quality data, i.e., raw data, and analysis was performed using BMKCloud (www.biocloud.net). In order to ensure the accuracy of information analysis, quality control of the original data is required to obtain a high-quality sequence, that is clean reads. Using miRanda and targetscan to predict miRNA targets, The targets of lncRNAs were predicted based on position relationship (within 100 kb of lncRNA) between lncRNA and mRNA. The source genes for circRNAs were obtained according to the position of the circRNA sequence; namely, the gene on which the circRNA sequence was located was regarded as its source gene.

### Quantitative validation

To verify the transcriptome analysis results, the selected lncRNAs, miRNAs, circRNAs, and mRNAs were tested by quantitative real-time polymerase chain reaction (qRT-PCR). Total RNA was extracted using the Trizol reagent (Invitrogen, CA, USA), and then reverse-transcribed into cDNA using TaKaRaPrimeScript RT reagent Kit with gDNA Eraser (RR047A, TaKaRa, Japan) according to the manufacturer’s instruction. RT-PCR was performed with SYBR Premix Ex Taq II (TaKaRa, Japan) and a PCR system (Illumina). The sequences of all primers are shown in Table 1. The data represent the means of three experiments.

**Table 1:** Primers of related genes in real time PCR

Name	Forward Primer	Reverse Rimer
miR-301a-3p	GCTACTGCTGACGACTGCTCTG	TGCTCCCGGATGCTTTGACAATAC
miR-7b	GCCAGAACACATGAGCCAATGC	ATGAGGCTGGCTGTGACTTGTTG
Hba-a2	CAATGACAGCTGCTCCAAGG	CAAGGGATCTCTGGAGGACC
Cd3e	TGAAAGCCAGAGTGTGCGAGAAC	GACCATCAGCAAGCCCAGAGTG
MSTRG.30797.2	CGTAGTCCGTAGGAGGTCGATC	GAAGAGAGATGTGACGCTGTGT
MSTRG.38795.2	AAGGCATTATAGGCTTGAACGG	ATACCTTGAAGACAACAGTGGCC
1:278,679,022|278,749,829	ACTTGTAAGATCTCCACGGTGA	TGGCAGCTCTAGGTTGATGA
11:34,612,627|34,631,134	GGTGACATCAATGGTTAAATCT	AGCCAGATAAACCAACGCATAT
GAPDH	CGGTGCTGAGTATGTCGTGGAG	GGTGGCAGTGATGGCATGGA

### GO and KEGG pathway analysis

To further investigate the potential function of the differentially expressed genes (DEGs), including differentially expressed mRNAs (DEmRNAs), source genes of differentially expressed circRNAs (DEcircRNAs), predicted target genes of differentially expressed miRNAs (DEmiRNAs), and predicted target genes of differentially expressed lncRNAs (DElncRNAs). The biological pathway and functional classification of these genes were performed using GO terms (http://geneontology.org/) and KEGG [17–19] pathways (http://genome.jp/kegg/). GO terms consisted of three components: biological process (BP), cellular component (CC), and molecular function (MF).

### Construction of co-expression network

Pearson correlation coefficient of genes were used to analysis the regulation work of ceRNA. ceRNA network were constructed and visually displayed using the Cystoscope software V3.5.0 (San Diego, CA, USA).

### Golgi staining

The brain tissue was stained by Golgi-Cox OptimStainTM Kit according to the manufacturer's protocol. We analysed the longest dendrite length, total length of dendrites, number of dendritic intersections, spinal density of the CA1, CA3 region using ImageJ 6.0 software (NIH, USA). Around 1000 spines or at least 1000 µm dendritic length were measured for each group. Calculate the density of dendritic spines which is the number of spines per µm dendrite [20]. Each rat selected three visual fields to measure the morphology quantitatively.

### Immunofluorescence

Each paraffin section was fixed with 4% paraformaldehyde, 0.25% Ttiton for 5 min, and blocked with 1% BSA. Then incubated with primary antibody overnight at 4 °C, including PSD95(1:500, cat#:20665-1-AP, Proteintech, China), SYN (1:300, cat#:67864-1-AP, Proteintech, China), MAP2(1:300, cat#:17490-1-AP, Proteintech, China). After washing, sections were incubated with conjugated secondary antibodies (1:250; Servicebio Co.,Ltd., China) in the dark for 60 min. Then nuclei were counterstained with DAPI (1:300, Servicebio Co.,Ltd., China) for 10 min. Images were photographed by confocal microscope (Leica TCS SP5 II). The fluorescent images were analyzed by Image J. In order to evaluate immunofluorescence, three visual fields of hippocampal CA1 and CA3 were randomly selected in each animal (*n* = 3 in each group).

### Western blotting

The total protein was extracted from hippocampal tissues using RIPA buffer. and measured by a bicinchoninic acid (BCA) protein kit (Bioss, China). The proteins were loaded onto 10% SDS-PAGE gel at 40 µg per lane for separation, and then transferred onto polyvinylidene fluoride (PVDF) membranes. The PVDF membrane were blocked with skimmed milk(5%) in Tris-buffered saline containing Tween at room temperature for 1 h, then incubated with primary antibodies against BDNF (1:200, cat#:28205-1-AP, Proteintech, China), TrkB (1:600, cat#:13129-1-AP, Proteintech, China), p- TrkB (1:1000, cat#:PA5-36695, Thermo Fisher, United States), PI3K (1:1200, cat#:20584-1-AP, Proteintech, China), Akt(1:50,000, cat#:Ab154598, Abcam, USA), p- Akt (1:10,000, cat#:66444-1-lg, Proteintech, China), β-actin at a dilution of 1/1000 at 4 °C overnight. Then, followed with secondary antibody (1:10,000, cat#: 611-145-002, Rockland, United States) at room temperature in the dark for 2 h. Finally, the protein bands were detected using the Odyssey imaging system (LICOR, United States).

### Statistical analysis

All data were analyzed using SPSS 20.0 and expressed as means ± SEM ($\overline x\pm\mathrm s$). *P*-value of 0.05 was considered statistically significant. The statistical analyses were performed by one-way analysis of variance (ANOVA) with least significant difference (LSD). tambane's T2 test was used when the variances were uneven.

## Results

### Qualitative and quantitative analysis of components in water extract of XYS

**Fig. 2:**
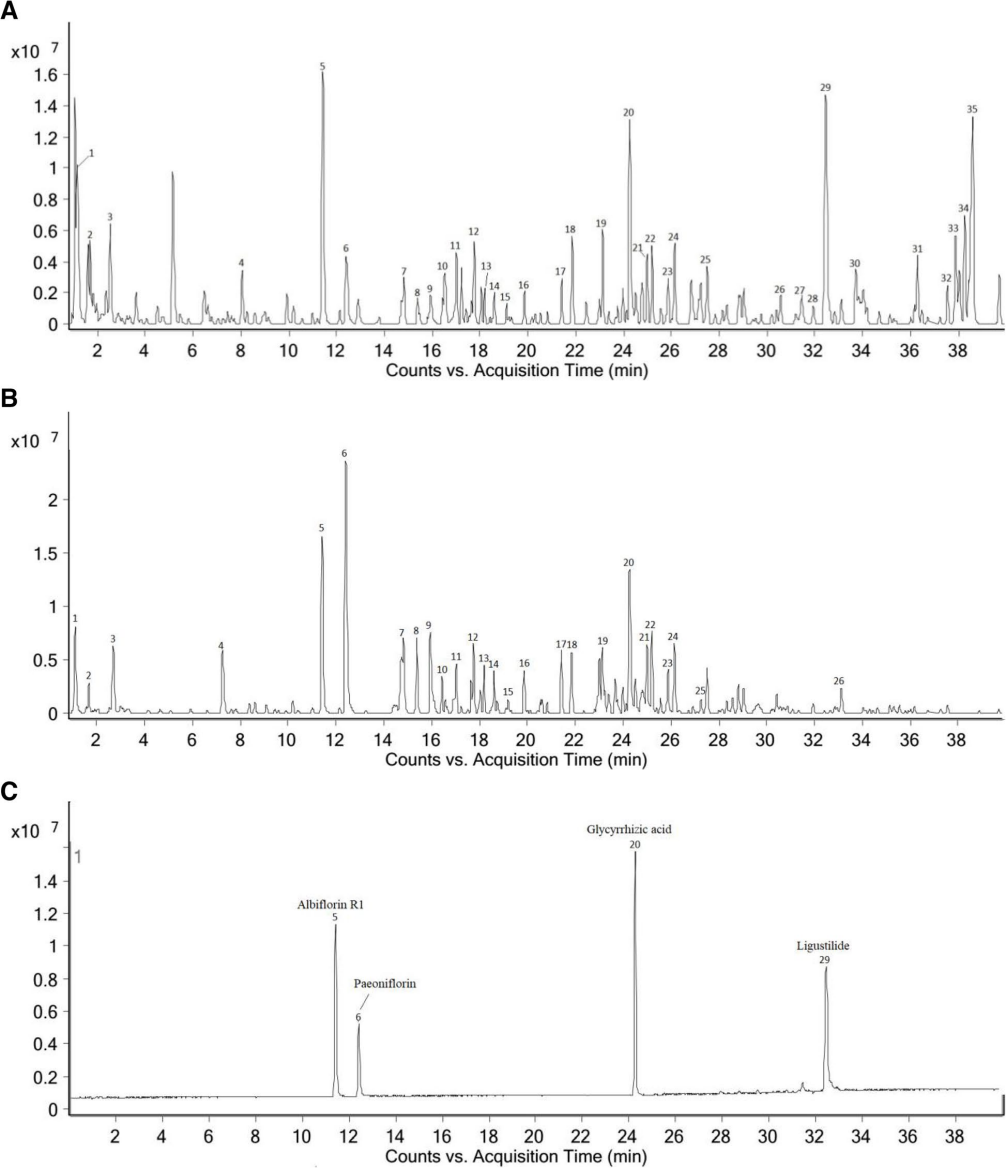
Identification of chemical components in water extract solutions of XYS by LC–MS/MS. **A** Base peak intensity chromatogram of LC–MS in water extract of Xiaoyao Wan in positive-ion mode. **B** Base peak intensity chromatogram of LC–MS in water extract of Xiaoyao Wan in negative-ion mode. **C** Base peak intensity chromatogram of LC–MS of reference substance in positive-ion mode

Qualitative analysis of XYS water extract was performed using LC–MS/MS. Chromatograms of the total ion in ESI negative and positive-ion mode for XYS water extract are shown in Fig. 2A and B. In the preliminary experiment, 35 compounds were identified in the extract, such as Citric acid, Adenosine, Albiflorin R1, Paeoniflorin, Benzoylpaeoniflorin, Licoricesaponine G, Liquiritin, etc. As shown in Fig. 2C, we chose four major compounds for further quantitative analysis according to the Pharmacopoeia of the People's Republic of China (2015 version). They were 3.55 mg/g Albiflorin, 1.46 mg/g Paeoniflorin, 4.89 mg/g Glycyrrhizic acid, and 6.14 mg/g Ligustilide in water extract of XYS.

### XYS ameliorates CUMS depression-like behaviors

**Fig. 3:**
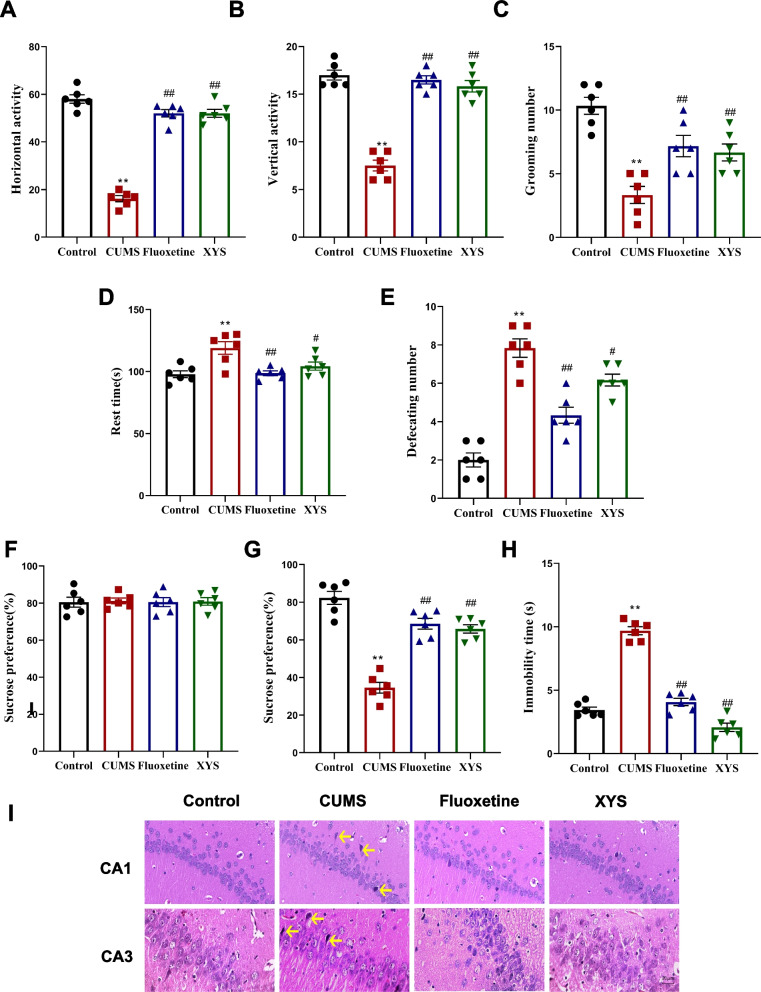
XYS ameliorate depressive-like behavior and hippocampal injury in CUMS rats. **A** Horizontal activity of the OF. **B** Vertical activity of the OF. **C** Grooming number. **D** Rest time. **E** Defecating number. **F** Sucrose preference (%). **G** Sucrose preference (%). **H** Immobility time(s) in FST. **I** Pathological changes in CA1 and CA3 area of hippocampal by HE staining (40x). Data are expressed as means ± SEM, *n* = 6 in each group. *P < 0.05; ** *P* < 0.01 vs. the control group; # *P* < 0.05; ## *P* < 0.01 vs. the CUMS group

In order to evaluate the antidepressant effect of XYS, we conducted OFT, SPT and FST. As shown in Fig. 3A, B, C the horizontal activity, vertical activity and grooming number were decreased in the CUMS group compared with the control group (*P* < 0.01), while after the treatment of XYS or fluoxetine, they were significant increased (both *P* < 0.01). As shown in Fig. 3D, E, compared with the control group, the rest time and defecating number were significantly increased in the CUMS group (*P* < 0.01), while XYS or fluoxetine significantly reduced the rest time and defecating number (*P* < 0.05 or *P* < 0.01). As for SPT shown in Fig. 3F, G, all rats exhibited similar baseline condition before CUMS (*P* > 0.05). A significant decrease in sucrose preference was noted in the rats after CUMS (*P* < 0.01). Nevertheless, XYS or fluoxetine increased sugar water preference significantly (both *P* < 0.01). The FST are shown in Fig. 3H, immobility time in the CUMS group were significantly longer compared with the control group (*P* < 0.01), and XYS or fluoxetine reduced immobility time in comparison with the CUMS group (both *P* < 0.01).

To further comfirm the protection of hippocampus by XYS, HE staining was used to observe the histopathological changes in the CA1, CA3 region. As shown in Fig. 3I, after exposed to CUMS, the structure of hippocampus was obviously damaged and the cells were pyknosist, the cell structure was disorganized, and the pyramidal cells and granulosa cells were damaged and disordered specifically. After the treatment of XYS or fluoxetine, only a few numbers of cells showed vacuolation and karyopyknosis, the hippocampal structures were basically intact, cells were in a more regular hierarchical structure, and the neuronal cell membrane was basically intact compared with CUMS group.

### The results of whole transcriptome analysis

#### Differentially expressed ncRNAs and mRNAs

**Fig. 4:**
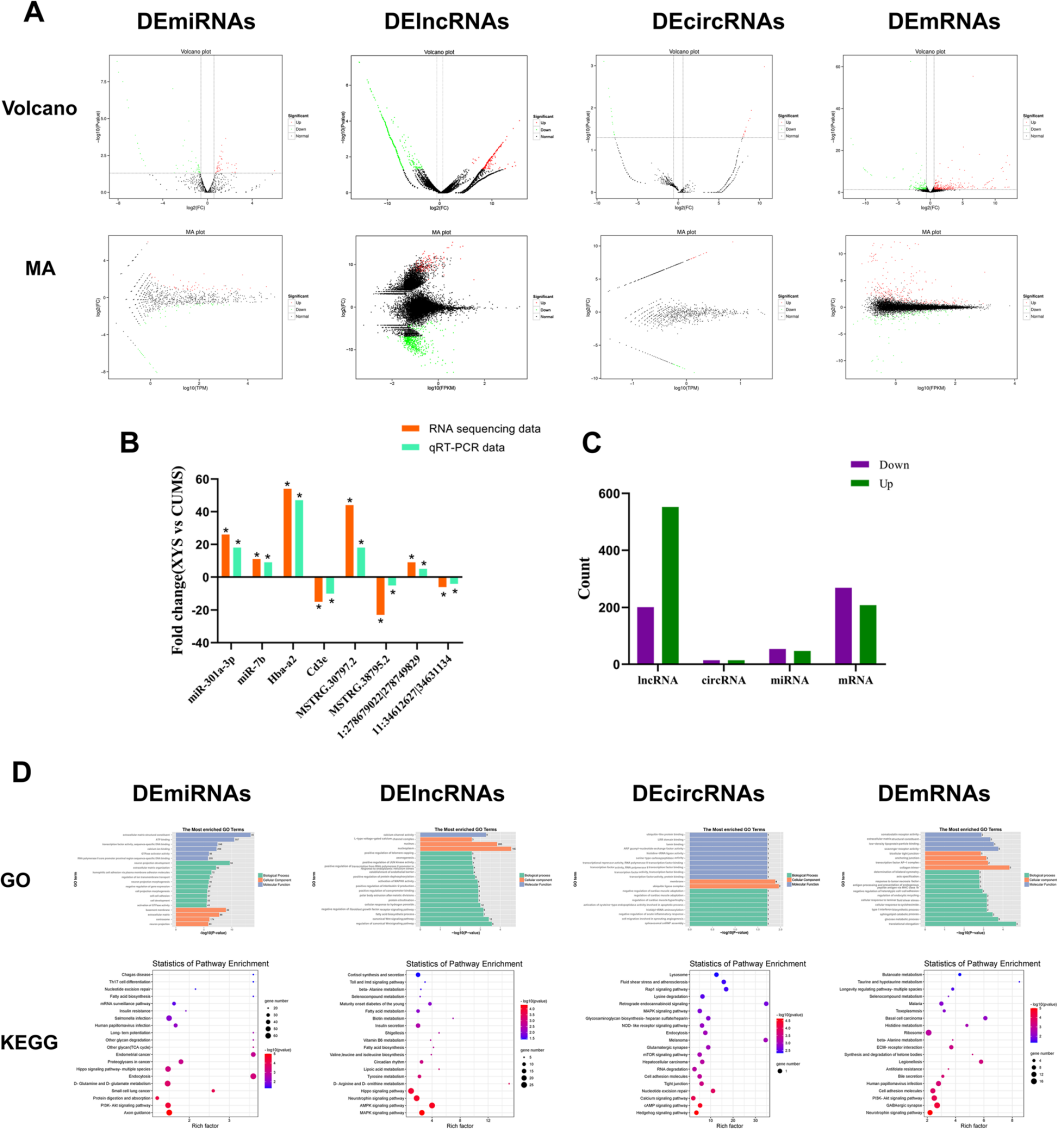
Differential expression analysis of miRNAs, lncRNAs, circRNAs and mRNAs by RNA-seq. **A** Volcano plot, MA plot of DEmiRNAs, DElncRNAs, DEcircRNAs and DEmRNAs expression profiles between XYS-treated CUMS rats and CUMS rats. **B** Number of up- and down-regulated ncRNAS and mRNAs. **C** The differential expression of ncRNAs and mRNAs were validated by qRT-PCR. **D** The GO, KEGG enrichment analysis of DElncRNA, DEmRNAs, DEmiRNAs, DEcircRNAs. In the GO analysis, the ontology contained biological process(BP), cellular component(CC), and molecular function(MF). *n* = 3 in each group. The abscissa indicates the number of genes, the ordinate indicates the GO term, and the color of the column indicates the corrected *p* value. In the KEGG analysis, the size of the dots represents the number of genes annotated in the pathway, and the color of the dots represents the corrected *p* value of the hypergeometric test

The expression of ncRNAs and mRNAs were detected by RNA sequencing in two groups (XYS-treated rats and CUMS rats). Information of the top 10 up-regulated and 10 down-regulated miRNAs, lncRNAs, circRNAs and mRNAs in the XYS-treated rats compared with the untreated rats are listed in Tables 1–4 (Supplementary tables). Volcano plot and MA plot were applied to exhibit DEncRNA and DEmRNA expression profiles between the XYS-treated rats compared with the untreated rats. Figure 4A indicates the volcano plot and MA plot of DEmiRNA, DElncRNA, DEcircRNA, and DEmRNA expression profiles, respectively. In total, our project detected 20,393 lncRNAs, 32,365 mRNAs, 1191 miRNAs, and 2195 circRNAs. As shown in Fig. 4B, there were 753 DElncRNAs (201 down-regulation and 552 up-regulation), 28 DEcircRNAs (14 down-regulation and 14 up-regulation), 101 DEmiRNAs (54 down-regulation and 47 up-regulation), and 477 DEmRNAs (269 down-regulation and 208 up-regulation) in the XYS-treated rats compared with the untreated rats respectively.

In order to verify the accuracy and reliability of transcriptome analysis results, 10 dysregulated ncRNAs and mRNAs were selected randomly for qRT-PCR analysis, including two miRNAs (rno-miR-301a-3p, rno-miR-7b), two mRNAs (Hba-a2, Cd3e), two lncRNAs (MSTRG.30797.2, MSTRG.38795.2), and two circRNAs (1:278679022|278749829 and 11:34612627|34631134). As shown in Fig. 4C, the results of sequencing data were consistent with the results of qRT-PCR.

#### GO and KEGG enrichment analysis

As shown in Fig. 4D, based on the GO enrichment analysis of targeted genes, the DEmiRNAs most significantly enriched were neuron projection development, basement membrane, and ATP binding; the DElncRNA most significantly enriched were canonical Wnt signaling pathway, nucleus, calcium channel activity; the DEcircRNAs significantly enriched were spliceosomal snRNP assembly, membrane, transcription factor activity and protein binding; the DEmRNAs most significantly enriched were translational elongation, collagen trimer, scavenger receptor activity, respectively.

The most significantly enriched KEGG pathways were also shown in Fig. 4D. For the DEmiRNAs, axon guidance, PI3K-Akt signaling pathway, and protein digestion and absorption were the most significant enriched pathway. For the DElncRNA, MAPK signaling pathway, AMPK signaling pathway, neurotrophin signaling pathway were the most significant enrichment pathways. For the DEcircRNA, Hedgehog signal pathway, cAMP signaling pathway, and Calcium signaling pathway were the most significant enriched pathway. For the DEmRNAs, neurotrophin signaling pathway, GABAergic synapse, PI3K-Akt signaling pathway were the most significant enriched pathway. These results suggest that these GO terms and KEGG pathways may contribute to the pathogenesis and biochemical characteristics of antidepressant effect of XYS. Therefore, we presumed that XYS exert antidepressant effect may be through different synapse or synaptic associated signal.

#### Regulatory network of ncRNAs and mRNA

**Fig. 5:**
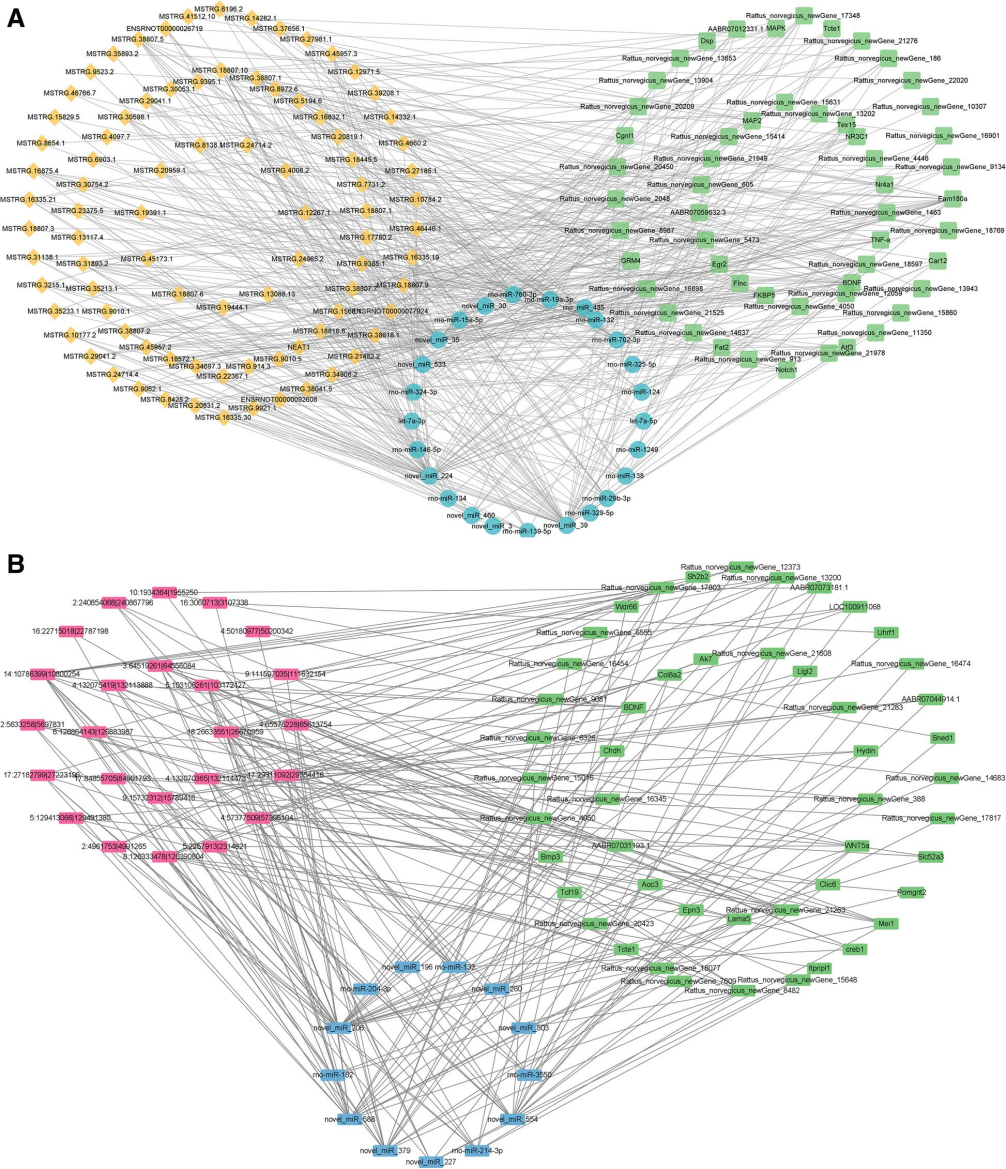
The network of ncRNAs and mRNA. **A** The interaction network of circRNA – miRNA – mRNA. **B** The interaction network of lncRNA–miRNA–mRNA

CircRNA, lncRNA, and mRNA could inhibit target gene regulation of miRNA, thus indirectly regulate gene expression and serving as a miRNA sponge to bind miRNA competitively with its binding sites, which was called competing endogenous RNA (ceRNA). To explore the molecular mechanism of ncRNAs, a circRNA–miRNA–mRNA regulatory network and lncRNA–miRNA–mRNA network were constructed based on the theory of ceRNA. As shown in Fig. 5A, the circRNA–miRNA–mRNA regulation network containing 49 mRNAs, 24 circRNAs, and 13 miRNAs was generated. As shown in Fig. 5B, the lncRNA–miRNA–mRNA regulation network that contained 54 mRNAs, 87 lncRNAs, and 25 miRNAs was built. These results suggested that circRNAs and lncRNAs harbor miRNA response elements and play pivotal regulatory roles in the mechanisms of XYS in anti-depression.

### XYS increased the density and number of dendrites and promoted the expression of MAP2 in CA1 and CA3 regions of hippocampus

**Fig. 6:**
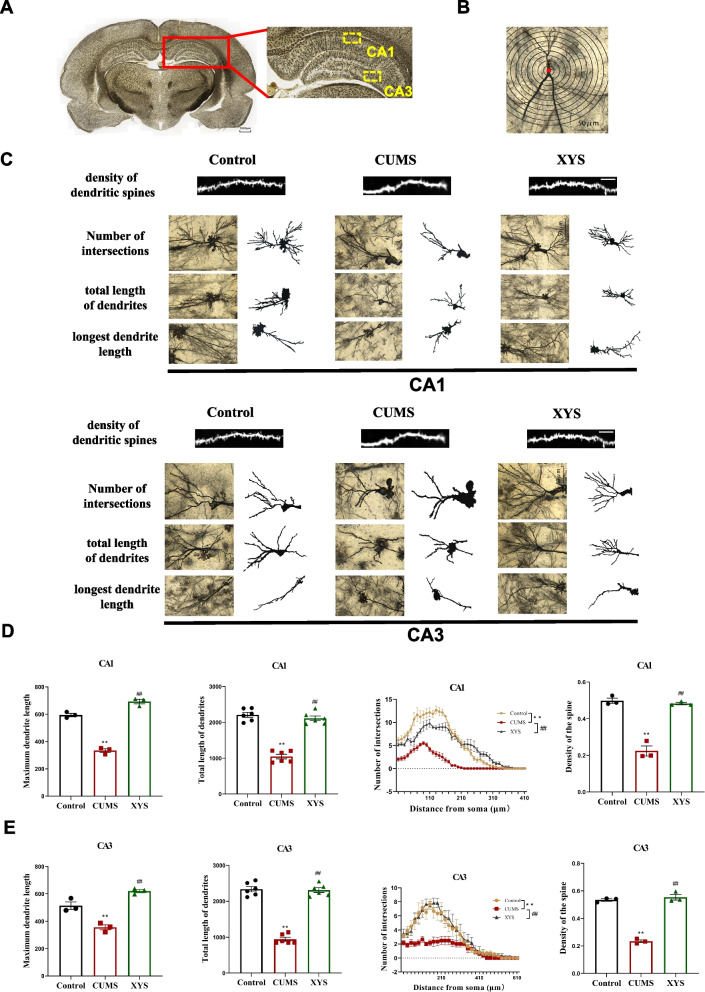
The morphological changes of synapses in the CA1, CA3 of hippocampal by Golgi staining. **A** Golgi staining of the whole brain, and each rat slice at the level of the CA1, CA3 region (box) (10x, 30x). **B** A CA3 neuron with the proximal portion of its apical dendrite (arrow), Sholl rings are placed at 20 um diameter intervals (200x). **C** Representative graphs of synapses in the CA1, CA3 of hippocampal neurons stained by Golgi (200x). **D** The longest dendrite length, total length of dendrites, the number of dendritic intersections, density of dendritic spines in each group of CA1 region. **E** The longest dendrite length, total length of dendrites, the number of dendritic intersections, density of dendritic spines in each group of CA3 region. Data are expressed as means ± SEM, *n* = 3 in each group. *P < 0.05; ** *P* < 0.01 vs. the control group; # *P* < 0.05; ## *P* < 0.01 vs. the CUMS group

As shown in Fig. 6, we used Golgi staining to observe synaptic morphological changes in specific regions of the hippocampus. In our results, the longest dendrite length, total length of dendrites, numbers of intersections, density of dendritic spines in the CA1, CA3 region of the hippocampus in the CUMS rats were significantly decreased compared with control group (*P* < 0.01). After the treatment of XYS, the longest dendrite length, total length of dendrites, numbers of intersections, and density of dendritic spines were all increased in the CA1, CA3 region of hippocampus (*P* < 0.01).

**Fig. 7:**
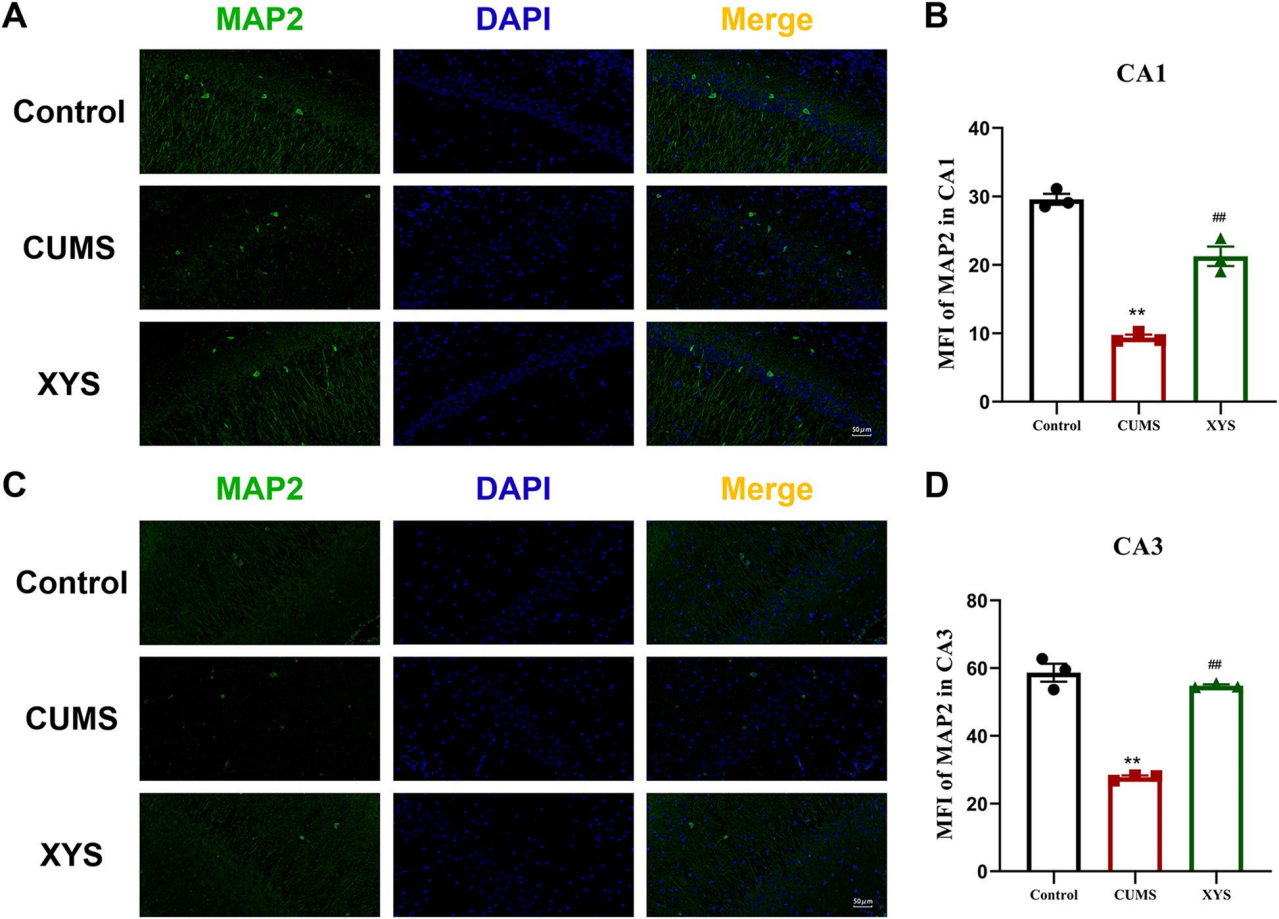
Effects of XYS on MAP2 in CA1, CA3 of hippocampal. **A** The immunofluorescence staining of MAP2 in CA1 (20x). **B** The expression of MAP2 in CA1. **C** The immunofluorescence staining of MAP2 in CA3 (20x). **D** The expression of MAP2 in CA3. Data are expressed as means ± SEM, *n* = 3 in each group. *P < 0.05; ** *P* < 0.01 vs. the control group; # *P* < 0.05; ## *P* < 0.01 vs. the CUMS group

In order to further explore the growth of axons and dendrites in the hippocampus, we analyzed the expression of skeletal proteins MAP2 in the CA1, CA3 regions of hippocampus. As shown in Fig. 7, the expression of MAP2 in the rat hippocampal CA1, CA3 areas in the CUMS groups were significantly decreased compared with the control group (*P* < 0.01). While, after the treatment with XYS, the MAP2 expression in the CA1, CA3 regions were significantly increased in the CUMS rats (*P* < 0.01).

### XYS increased the expression of PSD-95, synaptophysin in the hippocampal CA1, CA3 region of CUMS rats

**Fig. 8:**
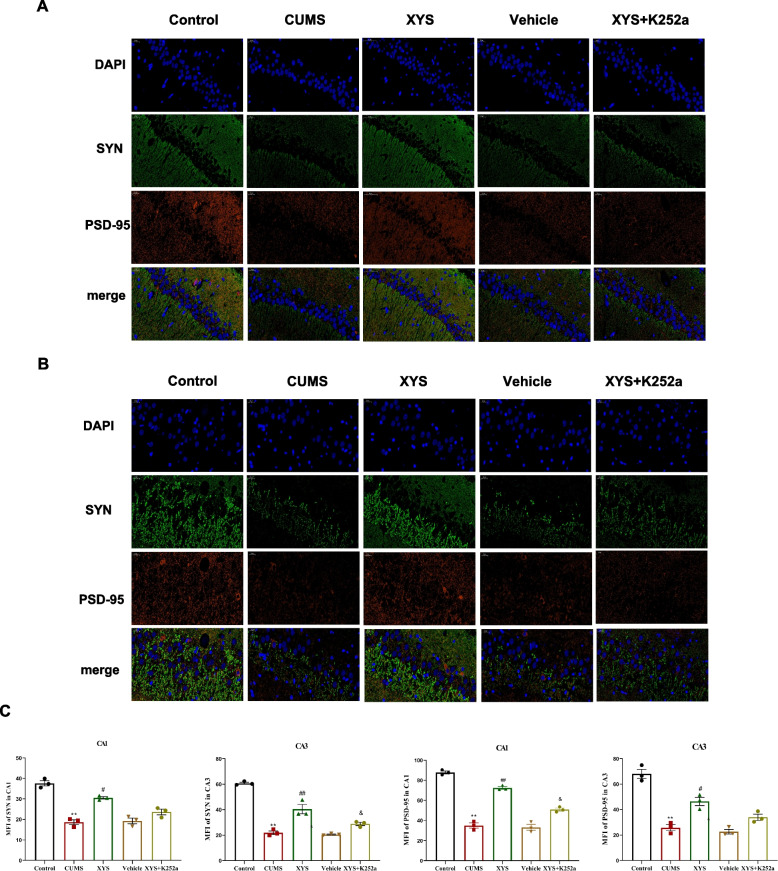
Effects of XYS on PSD-95, synaptophysin in CA1, CA3 of hippocampal. **A** The immunofluorescence staining of synaptophysin, PSD-95 in CA1 (40x). **B** The immunofluorescence staining of synaptophysin, PSD-95 in CA3 (40x). **C** The expression of synaptophysin in CA1. **D** The expression of synaptophysin in CA3. **E** The expression of PSD-95 in CA1. **F** The expression of PSD-95 in CA3. Data are expressed as means ± SEM, *n* = 3 in each group. *P < 0.05; ** *P* < 0.01 vs. the control group; ^#^ *P* < 0.05; ^##^ *P* < 0.01 vs. the CUMS group; ^&^ *P* < 0.05; ^&&^ *P* < 0.01 vs. the XYS group

Firstly, we examined the expression of PSD-95, synaptophysin in the CA1, CA3 region of hippocampal by immunofluorescence. As shown in Fig. 8, the expression of PSD-95, synaptophysin in the rat hippocampal CA1, CA3 areas of the CUMS were lower than that in the control group (*P* < 0.01), and the expression of PSD-95, synaptophysin was increased after the treatment of XYS compared with CUMS group (*P* < 0.05 or *P* < 0.01). Then, we used the trkB inhibitor k252a to further observe the regulatory mechanism of XYS on synapse loss. In our results, the expression of PSD-95, synaptophysin in the rat hippocampal CA1, CA3 areas of the vehicle group were lower than that in the control group (*P* < 0.01), and there was no difference between the rats in the CUMS and vehicle groups. However, after intrahippocampal microinjection of K252a, the expression of PSD-95, synaptophysin were decreased, which indicated that K252a reversed the protective effect of XYS on PSD-95 and synaptophysin. In addition, the expression of PSD-95 in CA1, and synaptophysin in CA3 were decreased in XYS + K252a group compared with XYS group simultaneously (*P* < 0.05).

### XYS increased the expression of PI3K, Akt through BDNF/trkB signal in CUMS rats

In order to explore the changes of growth and plasticity of axons and dendrites through the BDNF/trkB/PI3K signal, we use the trkB inhibitors K252a to inhibit the BDNF/trkB signal pathways to verify whether XYS could ameliorate synapse loss through BDNF/trkB/PI3K signal axis.

**Fig. 9:**
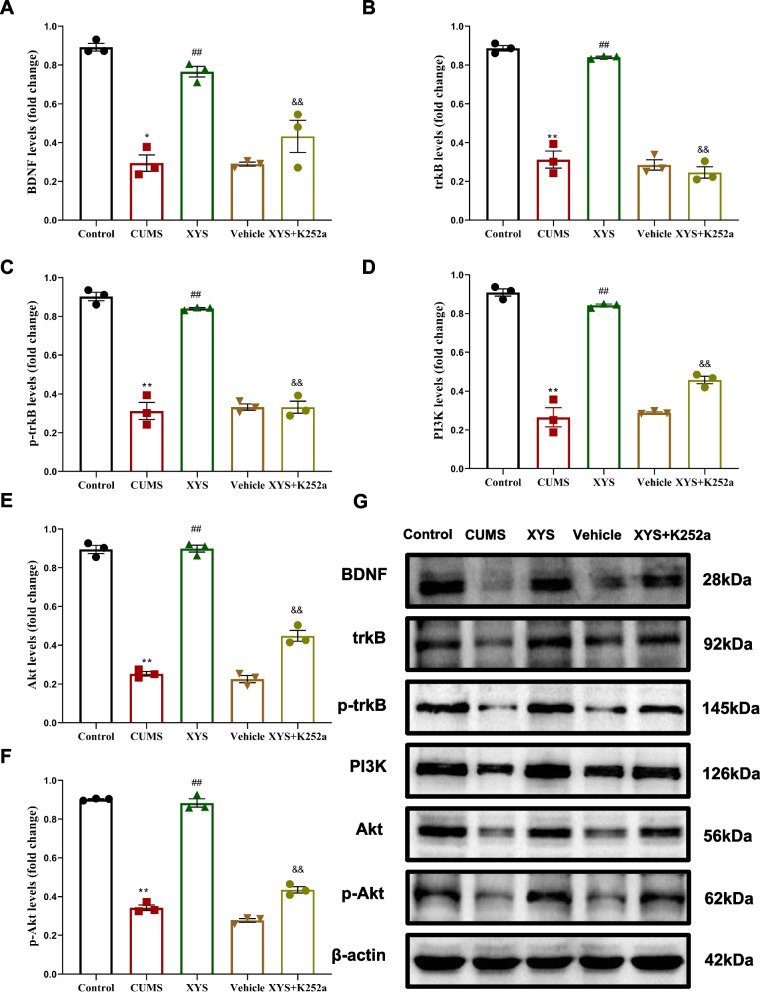
Effects of XYS on BDNF/trkB/PI3K signal axis in hippocampal. **A** The protein level of BDNF. **B** The protein level of trkB. **C** The protein level of p-trkB. **D** The protein level of PI3K. **E** The protein level of Akt. **F** The protein level of p-Akt. **G** Band diagram of Western blotting. Data are expressed as means ± SEM, *n* = 3 in each group. *P < 0.05; ** *P* < 0.01 vs. the control group; ^#^ *P* < 0.05; ^##^ *P* < 0.01 vs. the CUMS group; ^&^ *P* < 0.05; ^&&^ *P* < 0.01 vs. the XYS group

Further, we detected the level of BDNF, trkB, p-trkB. After exposed to CUMS, the level of BDNF, trkB, p-trkB in the hippocampal were decreased significantly compared with control group (*P* < 0.01 or *P* < 0.05). In addition, there was no difference between the rats in the CUMS and vehicle groups. As shown in Fig. 9A-C, XYS could increase the BDNF, trkB, p-trkB level of hippocampus in CUMS rats (*P* < 0.01), but the level of BDNF, trkB, p-trkB in hippocampus were decreased in XYS + K252a group compared with XYS group (*P* < 0.01).

Then, we detected the level of PI3K, Akt, p-Akt. After exposed to CUMS, the level of PI3K、Akt、p-Akt in the hippocampal were decreased significantly compared with control group (*P* < 0.01), and there was no difference between the rats in the CUMS and vehicle groups. As shown in Fig. 9D-F, XYS could increase the PI3K, Akt, p-Akt level of hippocampus in CUMS rats (*P* < 0.01), but the level of PI3K、Akt、p-Akt in hippocampus were decreased in XYS + K252a compared with XYS group (*P* < 0.01). The bands of BDNF, trkB, p-trkB, PI3K, Akt, p-Akt through Western blotting were shown in Fig. 9G.

## Discussion

Major depressive disorder (MDD) is a potentially life-threatening disease, which imposes a major public health and economic problem to the world [21]. XYS is one of the most commonly used antidepressants, but the exact mechanism of its anti-depressant effect remains unclear. As far as we know, this is the first comprehensive report of lncRNA, mRNA, circRNA, and miRNA to reveal regulator pathways with regard to XYS in CUMS rats.

Our study detected that there were 753 DElncRNAs, 28 DEcircRNAs, 101 DEmiRNAs and 477 DEmRNAs in the XYS-treated rats compared with the untreated rats respectively. Several DEmRNAs and DEmiRNAs may be connected with depression. However, the great majority of DElncRNAs and DEcircRNAs were unknown, mainly owing to few related studies. In addition, 10 dysregulated ncRNAs and mRNAs identified were selected for qRT-PCR validation, the results confirmed the sequencing analysis findings to some extent. The effects of XYS on ncRNAs, including miRNAs, lncRNAs, and circRNAs were evaluated in our study. Reported evidence has demonstrated that after CUMS, the expression of miR-132, miR-138, miR-191 and miR-146-5p in hippocampus of SD rats is down-regulated, while the miR-485, miR-124, miR-139-5p, miR-29b-3p were up-regulated [22]. In our study, XYS can effectively interfere with the expression of these miRNAs. Previous report have found that miR-132 can regulated spine formation [23], miR-485 closely associated with synaptic structural plasticity [24]. miR-191, miR-192-5p can affect the expression of synapse-associated proteins and quantity of synaptic structures (dendrites and spines) [25]. Furthermore, some DEmiRNAs, such as novel-miR-12059, novel-miR-605, novel-miR-2048, and novel-miR-5701 were also found to be significantly different after the treatment of XYS, which suggested that these miRNAs play an important role in the anti-depressant mechanism of XYS. These genes were firstly reported related to depression and might deserve further study.

miRNAs and their translation proteins in neuronal dendrites and axons promote the generation and maintenance of synapses. Mature miRNA and pre-miRNA located in neurons are involved in the regulation of synaptic-related protein synthesis. From the result of lncRNA-miRNA-mRNA network, LncRNA NEAT1 were identified to binding miRNA-124 which target BDNF/TrkB signal regulating synaptogenesis and neuronal proliferation [26]. Meanwhile, circRNAs can act as endogenous sponges to influence miRNA activity, and affect mRNA by interacting with the Pol II complex in the nucleus [27]. Knockdown of CNTNAP2 resulted in decreasing of neurite length, while circ-0087100 can target on CNTNAP3 in the depression [28]. In addition, KEGG analysis revealed that circRNA, lncRNA, miRNA, mRNA were mainly enriched in different synapses or synaptic-related protein, such as neurotrophin signaling pathway, glutamatergic synapse, GABAergic synapse. Depression involves a combination of genetic susceptibility, epigenetic susceptibility and environmental risk factors, but no single gene candidate gene has a strong enough effect to provide a convincing mechanism hypothesis [29]. Therefore, most studies believe that depression is a complex and heterogeneous set of symptoms caused by multiple gene, and each has little impact on risk, that converges to a common circuit, cellular and molecular pathway finally [30]. Therefore, our whole transcriptome study provides a good basis for XYS exerting antidepressant effect, which focused on synapse.

In the study of pathogenesis about depression, synaptic loss led to symptoms associated with depressive disorder [31]. Sustained high levels of stress are thought to cause synaptic loss of potential circuits in emotional and cognitive processes [32]. In order to further explore the antidepressant mechanism of XYS, the potential targets and pathways of XYS were predicted by GO biological function analysis and KEGG pathway enrichment. Our results showed that neurotrophin signaling and PI3K/Akt pathway were participated by mRNA, miRNA, lncRNA. Previous studies have reported that these two signaling pathways are closely related to the pathogenesis of depression [33]. The most data of neurotrophin hypothesis concerns BDNF and its receptor trkB, which might contribute to the occurrence of depression through atrophy of synapse [34]. Several studies confirmed that BDNF/trkB signal plays an important role in dendritic growth, axonal and dendritic arborization and pruning, spine density, maturation and stabilization [35, 36]. mBDNF binds with the high-affinity trkB receptor, then phosphorylated-trkB activates several enzymes, such as PI3K, mitogen-activated protein kinase (MAPK), and guanosine triphosphate hydrolases (GTP-ases) [37]. New, supernumerary and fully functional synapses are mediated by the PI3K/Akt signal [38], and PI3K/Akt cascade could enhances dendritic growth and branching through regulation of protein synthesis and cytoskeleton development [39]. Hence, our whole transcriptome analysis and prediction confirm that the antidepressant effect of XYS may be closely related to synaptic loss.

Many studies have found that hippocampal volume reduction and synaptic deficits in functional connectivity are considered to be related to the pathogenesis of depression [40, 41]. Meanwhile, previous evidence shows that the number and function of synapses in patients with depression are reduced [42]. Dendritic spines in the apical dendrites in the hippocampal CA3 and CA1 regions are more sensitive to stress [43], and CUMS could induce synaptic spine loss in the hippocampus CA3, CA1 region [44]. Moreover, losses of spine number and dendritic arbor in hippocampus were observed in rats exposed to chronic stress [45], these findings suggest that synapse loss is a key indicator of depression and depression like behavior. Golgi staining is used to observe the morphological changes of axons, dendrites and dendritic spines. Our study found that the longest dendrite length and total length of dendrites were shorter, and the density of dendritic spines was decreased in the CA1, CA3 region of the hippocampus after exposed to the CUMS. Chronic stress induces changes in synaptic function that occur in parallel with the atrophy of distal apical dendritic branches in pyramidal cells and decreases in the number of dendritic spines [46], and our research is consistent with their results. XYS is proven a safe and effective treatment or adjuvant therapy for depression [47], which could regulated necroptosis [8], alleviates hippocampal glutamate-induced toxicity [9], regulating autophagy in hypothalamic neuron [48], modulated the gut microbiota and regulating the NLRP3 inflammasome [49]. However, there are few reports on the antidepressant effect of XYS with synapse. In our result, we found that the treatment of XYS could significantly extend the longest dendrite length, total length of dendrites, also it could increase the number of dendritic intersections and density of dendritic spines in the CA1, CA3 region of hippocampus, which further confirmed the protective effect of XYS on synapses loss.

Axon is guided by a moving structure named "growth cone", which responds to different molecular clues, such as attraction and repulsion, while the dynamic cytoskeleton of microtubule in the "growth cone" play a major role in axonal growth [50]. MAP2 is a specific microtubule-associated protein used as a marker of synapse [51], and the level of MAP2 in hippocampus were lower than control has been confirmed by depressive patients and CUMS rats [52, 53]. While XYS could increase the expression of the MAP2 protein in CUMS and corticosterone-induced stress rats [9, 54]. The results of other studies are consistent with our results. We found that the expression of MAP2 in the CUMS rats of hippocampal CA1, CA3 region were reduced, while XYS promoted its expression in CA1 and CA3 regions of hippocampus, which indicates that XYS can promote synapse growth.

Many proteins expressed in presynaptic terminals and postsynaptic density are used as markers of synaptic density. Synaptophysin is an accurate index of neuronal synaptic density mainly expressed in the presynaptic nerve endings, which interacts with synaptobrevin, thus participating in synaptic vesicle exocytosis [55]. Synaptophysin is integral to the synaptic vesicle membrane, which could be used in the quantification of synapses consequently [56]. PSD-95 (postsynaptic density protein 95) is abundant in the postsynaptic density (PSD), which is implicated in regulating synaptic strength and plasticity, and in forming and maintaining excitatory synapses [57]. In order to evaluate synapse loss in the hippocampus, we tested the presynaptic marker synaptophysin and postsynaptic marker PSD-95 respectively. In our study, we found that the expression of synaptophysin and PSD-95 were decreased significantly in the CUMS rats, while after the treatment of XYS, the expression of synaptophysin and PSD-95 were increased. Also, we found that there is no difference between CUMS and vehicle group. However, the advancing effect of XYS on synapse were blocked after hippocampal microinjection trkB inhibitor K252a, which further proves that XYS raise core synaptic protein may be through BDNF/trkB signal. BDNF acts as paracrine and autocrine factor on both pre-synaptic and post-synaptic target sites [58]. BDNF/TrkB related downstream molecular pathways include PI3K, ERK, and MAPK signals. Interestingly, dysregulation of these pathways could regulate the levels of essential synaptic proteins, such as PSD-95 and synaptophysin [59, 60]. Therefore, these findings may indicate that the antidepressant effect of XYS in CUMS rats is driven by the improved expression of PSD-95 and synaptophysin in CA1, CA3 regions via BDNF/trkB signal, resulting in an increased synaptic strength and enhanced synaptic growth.

BDNF, which is required for the action of antidepressants [61], is one of the most studied neurotrophic proteins that efficiently modify synaptic strength, dendritic spines as well as synaptic plasticity [62]. When mature BDNF bind to the extracellular domain of trkB, the intracellular domains of the receptor dimerize and autophosphorylate one of three tyrosine residues, and initiates a distinct signaling cascade, such as PI3K/Akt, MAP kinase/Erk, or phospholipase C, resulting in synaptogenesis and synaptic plasticity [63]. Hence, BDNF is supposed as a major active substance which could activate PI3K/Akt signal [64]. In our study, we found that BDNF, trkB, p-trkB, PI3K, AKT, p-AKT were decreased in CUMS group, while XYS could increase the expression of BDNF, trkB, p-trkB, PI3K, AKT, p-AKT, which further proved that XYS can adjust BDNF/trkB/PI3K signal axis in hippocampal. BDNF binds with TrkB receptor, phosphorylated-TrkB activates the PI3K/Akt cascade, then enhances dendritic growth and branching through regulating protein synthesis and cytoskeleton development [65]. Inhibited BDNF/trkB signaling display a depression-like behavior, with decreasing length of dendrites and dendritic spine density in CA1 pyramidal neurons [66]. In order to further analyze the effect of XYS on synaptic loss, we used the inhibitor of TrkB, which is the intermediate junction of neurotrophic signal and PI3K/Akt signal. After microinjection in hippocampus of K252a, a trkB specific inhibitor, the expression of trkB, p-trkB were reversed after XYS treatment. Previous research have shown that PI3K involved in synapse formation in drosophila [67], and new, redundant and fully functional synapses were promoted by PI3K signal with age-independent [68]. In addition, the regulation of BDNF/trkB expression can trigger PI3K pathway. Meanwhile, the expression of PI3K, AKT, p-AKT were decreased after the treatment of XYS accompanied by K252a, which further indicate that XYS exert antidepressant effect may be through BDNF/trkB/PI3K signal axis.

## Conclusion

For the first time, our research based on ceRNA network further exploring the antidepressant mechanism of XYS. DEmRNAs and DEncRNAs in the coding and non-coding transcriptomes of hippocampus in CUMS were identified, and combining it with bioinformatics analysis may provide a better understanding of the potential roles of miRNA, lncRNA, circRNA, and mRNA in depression. Moreover, our strategy found that the effect of XYS on reducing hippocampal synapse loss in CUMS rats may be related to the activation of BDNF/trkB/PI3K signal axis. Nevertheless, this study is far from sufficient, and more investigations are needed to be further explored.

## Supplementary Information

**Additional file 1: Table 1. **The top 10 up-and down-regulated miRNAs. **Table 2.** The top 10 up-and down-regulated lncRNAs. **Table 3.** The top 10 up-and down-regulated circRNAs. **Table 4.** The top 10 up-and down-regulated mRNAs.

**Additional file 2.**

## Data Availability

The original contributions presented in the study are included in the article/supplementary material, further inquiries can be directed to the corresponding authors.
