# Supplementary material for: A whole transcriptome profiling analysis for antidepressant mechanism of Xiaoyaosan mediated synapse loss via BDNF/trkB/PI3K signal axis in CUMS rats
Source: BMC Complement Med Ther. 2023 Jun 15;23:198. doi: 10.1186/s12906-023-04000-0 (PMC10273699; doi:10.1186/s12906-023-04000-0)
Supplement: Supplementary file 1 — Additional file 1: Table 1. The top 10 up-and down-regulated miRNAs. Table 2. The top 10 up-and down-regulated lncRNAs. Table 3. The top 10 up-and down-regulated circRNAs. Table 4. The top 10 up-and down-regulated mRNAs. [file 12906_2023_4000_MOESM1_ESM.docx]

**Tab.1 The top 10 up-and down-regulated miRNAs.**

| Name | XYS1 | XYS2 | XYS3 | CUMS1 | CUMS2 | CUMS3 | Pvalue | log2FC | regulated |
| --- | --- | --- | --- | --- | --- | --- | --- | --- | --- |
| rno-miR-451-5p | 750.0863 | 641.1452 | 670.2109 | 234.2926 | 181.1802 | 201.2457 | 2.86E-06 | 1.9122 | up |
| rno-miR-186-3p | 76.5836 | 109.0915 | 90.4532 | 32.4134 | 35.2476 | 39.7439 | 0.000221 | 1.6269 | up |
| rno-miR-320-3p | 1248.3721 | 1371.1831 | 1405.2947 | 585.0856 | 607.1276 | 725.3659 | 0.000650 | 1.2692 | up |
| rno-miR-365-3p | 65.3618 | 65.6323 | 66.1697 | 30.0646 | 31.0027613 | 30.4593 | 0.001642 | 1.2287 | up |
| rno-miR-301a-3p | 88.9866 | 151.6639 | 140.4376 | 50.3817 | 76.9432 | 67.4532 | 0.001838 | 1.3666 | up |
| novel_miR_157 | 103.358 | 100.2223 | 104.3598 | 51.9084 | 52.7965 | 54.6432 | 0.003536 | 1.0768 | up |
| rno-miR-7a-5p | 77211.0567 | 63899.4427 | 72396.1877 | 33173.2509 | 31659.2764 | 30080.4593 | 0.003757 | 1.1929 | up |
| novel_miR_224 | 44.8870 | 35.4769 | 39.7584 | 18.3206 | 20.3964 | 21.3764 | 0.005245 | 1.2354 | up |
| rno-miR-10a-5p | 102.3739 | 59.9560 | 77.4597 | 35.9366 | 40.8764 | 42.3579 | 0.005344 | 1.2755 | up |
| novel_miR_359 | 23.6247 | 31.9292 | 30.9867 | 12.2137 | 14.8576 | 10.6055 | 0.005791 | 1.2935 | up |
| novel_miR_167 | 0.2581 | 0 | 0.3557 | 5.5196 | 4.6754 | 4.3276 | 1.33E-09 | -8.0919 | down |
| novel_miR_356 | 5.0499 | 5.2765 | 4.8763 | 0.2581 | 0 | 0.2257 | 7.16E-09 | -7.9640 | down |
| novel_miR_281 | 0.1094 | 0.1763 | 0.2057 | 3.8755 | 4.0985 | 4.0025 | 3.12E-08 | -7.5839 | down |
| rno-miR-881-3p | 1.3781 | 0.8869 | 1.1765 | 10.5695 | 9.8766 | 9.4223 | 9.27E-08 | -3.1076 | down |
| rno-miR-741-3p | 0 | 0.2459 | 0.2981 | 2.9359 | 3.2764 | 3.5276 | 5.54E-07 | -6.9858 | down |
| novel_miR_126 | 0.1976 | 0.2122 | 0.2099 | 2.7011 | 2.9433 | 2.6431 | 1.85E-06 | -7.0664 | down |
| rno-miR-122-5p | 20.0810 | 24.6564 | 23.3276 | 76.4533 | 79.4483 | 72.1964 | 1.47E-05 | -1.6635 | down |
| rno-miR-871-3p | 0.2288 | 0.2135 | 0.1543 | 1.7615 | 2.0943 | 2.1479 | 0.0001 | -6.4554 | down |
| rno-miR-6215 | 8.4556 | 8.1447 | 7.8325 | 2.5593 | 1.2416 | 1.8553 | 0.000297 | -2.05435 | down |
| rno-miR-743b-3p | 1.5267 | 1.7491 | 1.8943 | 0.2177 | 0.1639 | 0.2159 | 0.000463 | -6.2515 | down |

**Tab.2 The top 10 up-and down-regulated lncRNAs.**

| Name | XYS1 | XYS2 | XYS1 | CUMS1 | CUMS2 | CUMS3 | Pvalue | log2FC | regulated |
| --- | --- | --- | --- | --- | --- | --- | --- | --- | --- |
| ENSRNOT00000093026 | 6.4011 | 5.9165 | 6.1246 | 0 | 0 | 0 | 9.32E-05 | 15.2628 | up |
| MSTRG.31606.3 | 12.2027 | 14.2635 | 11.0409 | 0 | 0 | 0 | 0.000262 | 14.0971 | up |
| MSTRG.4097.7 | 2.8386 | 2.2864 | 3.0475 | 0 | 0 | 0 | 0.000729 | 12.8601 | up |
| MSTRG.30598.1 | 0.5176 | 0.1835 | 0.4761 | 0 | 0 | 0 | 0.001149 | 12.6466 | up |
| MSTRG.16875.4 | 0.6341 | 0.6574 | 0.7124 | 0 | 0 | 0 | 0.001558 | 12.1312 | up |
| MSTRG.6228.1 | 0.5941 | 0.5124 | 0.6014 | 0 | 0 | 0 | 0.001569 | 12.1092 | up |
| MSTRG.24654.1 | 1.3844 | 1.1094 | 1.5791 | 0 | 0 | 0 | 0.001742 | 11.9725 | up |
| MSTRG.19436.9 | 1.0041 | 0.9232 | 0.8439 | 0 | 0 | 0 | 0.002062 | 11.7952 | up |
| MSTRG.16166.1 | 0.6662 | 0.4784 | 0.7124 | 0 | 0 | 0 | 0.002068 | 11.8329 | up |
| MSTRG.46529.1 | 1.3395 | 0.8124 | 1.0954 | 0 | 0 | 0 | 0.002222 | 11.7377 | up |
| ENSRNOT00000057108 | 7.0435 | 6.8549 | 6.7320 | 0 | 0 | 0 | 5.03E-08 | -15.4215 | down |
| MSTRG.7136.1 | 13.4708 | 14.8596 | 13.8744 | 0 | 0 | 0 | 5.35E-08 | -15.3528 | down |
| MSTRG.25591.5 | 0.5565 | 1.1439 | 0.9862 | 0 | 0 | 0 | 5.00E-07 | -13.8760 | down |
| MSTRG.45173.1 | 0.8041 | 1.3946 | 1.1394 | 0 | 0 | 0 | 6.42E-07 | -13.6833 | down |
| MSTRG.23280.2 | 4.3301 | 4.6954 | 4.1259 | 0 | 0 | 0 | 7.36E-07 | -13.6504 | down |
| MSTRG.12513.1 | 6.0225 | 5.9645 | 5.5943 | 0 | 0 | 0 | 1.55E-06 | -13.2979 | down |
| MSTRG.20634.1 | 2.3047 | 2.4963 | 2.7411 | 0 | 0 | 0 | 1.71E-06 | -13.2455 | down |
| MSTRG.13088.13 | 1.370493 | 1.521764 | 1.895739 | 0 | 0 | 0 | 2.05E-06 | -13.1127 | down |
| MSTRG.22668.1 | 12.063004 | 12.543923 | 11.954227 | 0 | 0 | 0 | 2.31E-06 | -13.0452 | down |
| MSTRG.7320.1 | 2.818563 | 2.946634 | 3.479652 | 0 | 0 | 0 | 3.41E-06 | -12.7834 | down |

**Tab.3 The top 10 up-and down-regulated circRNAs.**

| Name | XYS1 | XYS2 | XYS1 | CUMS1 | CUMS2 | CUMS3 | Pvalue | log2FC | regulated |
| --- | --- | --- | --- | --- | --- | --- | --- | --- | --- |
| 16:23555919\|23600154 | 0 | 0 | 0 | 11.1178 | 10.4273 | 10.9675 | 0.001056 | 10.6120 | up |
| 13:95167819\|95208329 | 0 | 0 | 0 | 3.4385 | 3.6198 | 3.8374 | 0.011455 | 9.0031 | up |
| 8:23508992\|23560371 | 0 | 0 | 0 | 3.8396 | 3.1335 | 3.4269 | 0.011470 | 8.9875 | up |
| 11:10020678\|10022991 | 0 | 0 | 0 | 3.2092 | 3.0255 | 3.1756 | 0.017755 | 8.8252 | up |
| 18:29633808\|29634585 | 0 | 0 | 0 | 2.4069 | 3.0795 | 2.8965 | 0.019913 | 8.6390 | up |
| 14:37228632\|37254001 | 0 | 0 | 0 | 2.2923 | 2.1611 | 2.0845 | 0.028683 | 8.3411 | up |
| 1:247944245\|247960553 | 0 | 0 | 0 | 2.0631 | 2.0530 | 2.0794 | 0.033913 | 8.2274 | up |
| 4:66214543\|66219277 | 0 | 0 | 0 | 1.8338 | 2.1611 | 2.0741 | 0.036187 | 8.1832 | up |
| 4:67299951\|67301045 | 0 | 0 | 0 | 1.3754 | 2.8094 | 1.7869 | 0.036442 | 8.2462 | up |
| 8:84434784\|84496086 | 0 | 0 | 0 | 2.9227 | 1.2426 | 2.1765 | 0.036801 | 8.2503 | up |
| 10:107845994\|107850764 | 4.1820 | 3.7465 | 3.9554 | 0 | 0 | 0 | 0.000792 | -9.1479 | down |
| 6:14755038\|14758647 | 2.7881 | 2.9463 | 3.0429 | 0 | 0 | 0 | 0.003425 | -8.5643 | down |
| 8:28018432\|28026404 | 2.5556 | 2.6345 | 2.7599 | 0 | 0 | 0 | 0.005482 | -8.4391 | down |
| 2:188269009\|188286112 | 2.2652 | 2.3954 | 2.5861 | 0 | 0 | 0 | 0.010301 | -8.2656 | down |
| 1:162684057\|162696070 | 2.2071 | 2.4459 | 2.3562 | 0 | 0 | 0 | 0.012080 | -8.2282 | down |
| X:82845576\|82846306 | 2.0511 | 2.3506 | 2.2984 | 0 | 0 | 0 | 0.015943 | -8.1505 | down |
| 8:82038973\|82045524 | 2.0910 | 2.2214 | 2.1765 | 0 | 0 | 0 | 0.016177 | -8.1387 | down |
| 17:24032670\|24045965 | 2.0329 | 2.2957 | 2.5126 | 0 | 0 | 0 | 0.021883 | -8.1100 | down |
| 2:224802843\|224803455 | 1.9748 | 2.0906 | 2.1662 | 0 | 0 | 0 | 0.024177 | -8.0683 | down |
| 9:28529457\|28534987 | 1.8005 | 1.9507 | 2.1439 | 0 | 0 | 0 | 0.037234 | -7.9356 | down |

**Tab.4 The top 10 up-and down-regulated mRNAs.**

| Name | XYS1 | XYS2 | XYS1 | CUMS1 | CUMS2 | CUMS3 | Pvalue | log2FC | regulated |
| --- | --- | --- | --- | --- | --- | --- | --- | --- | --- |
| LOC103694857 | 96.4527 | 81.7321 | 102.5294 | 305.1059 | 276.9281 | 314.2496 | 3.69E-21 | 1.7558 | up |
| AABR07002779.1 | 0.0669 | 0.0787 | 0.1439 | 9.5651 | 10.3766 | 11.6571 | 5.16E-18 | 6.6605 | up |
| LOC103694855 | 59.2534 | 48.9220 | 62.2767 | 139.2565 | 144.9591 | 142.7759 | 5.44E-13 | 1.3332 | up |
| Hba-a2 | 154.5021 | 127.7878 | 139.6655 | 363.0830 | 374.1965 | 380.1865 | 2.61E-12 | 1.3430 | up |
| LOC103690996 | 1.0459 | 1.6619 | 1.9641 | 23.0745 | 20.1765 | 21.9216 | 4.12E-11 | 4.0537 | up |
| Hba-a1 | 354.8552 | 319.2561 | 369.2788 | 769.3606 | 801.1973 | 787.5546 | 5.96E-10 | 1.1706 | up |
| Sstr1 | 0.1438 | 0.1199 | 0.1791 | 1.4843 | 1.6755 | 1.8291 | 1.80E-08 | 3.4501 | up |
| AABR07025010.1 | 45.7741 | 25.5759 | 30.5629 | 88.3427 | 90.5761 | 95.8924 | 1.01E-06 | 1.2877 | up |
| AABR07018038.2 | 0.0443 | 0.0628 | 0.0971 | 0.5868 | 0.6344 | 0.6694 | 7.38E-06 | 3.4165 | up |
| Ifi27 | 15.1552 | 15.6775 | 16.1643 | 38.3585 | 40.5674 | 43.1924 | 7.59E-06 | 1.2942 | up |
| AABR07054368.1 | 97.5443 | 66.6118 | 79.6257 | 8.4585 | 9.7169 | 11.1507 | 1.83E-32 | -3.2967 | down |
| AABR07011951.1 | 35.1247 | 21.7584 | 27.4479 | 3.4150 | 4.2188 | 3.8576 | 2.40E-23 | -3.0753 | down |
| AABR07028352.1 | 5.2659 | 8.8894 | 7.1654 | 0.3115 | 0.44715 | 0.7159 | 9.39E-18 | -4.5171 | down |
| RGD1359290 | 21.8834 | 17.9409 | 19.1576 | 2.7054 | 3.2177 | 3.5419 | 5.35E-09 | -2.8949 | down |
| Hba-a3 | 4.7613 | 3.9117 | 4.2266 | 0 | 9.8767 | 10.9564 | 3.30E-08 | -10.1118 | down |
| Bmp4 | 5.7134 | 5.0676 | 5.8926 | 0.6849 | 0.7542 | 0.8124 | 6.03E-08 | -2.9858 | down |
| Igf2 | 13.5095 | 27.5165 | 18.9453 | 6.2721 | 5.2418 | 4.9238 | 3.98E-07 | -1.7607 | down |
| LOC103689961 | 148.8138 | 190.3962 | 210.6649 | 82.6692 | 64.0223 | 91.5868 | 9.71E-07 | -1.0567 | down |
| Nmur2 | 0.7329 | 0.8781 | 0.9552 | 0.0426 | 0.0655 | 0.0559 | 2.75E-06 | -4.1557 | down |
| Fkbp9 | 18.4257 | 18.8858 | 21.3376 | 10.6556 | 12.8765 | 13.1926 | 4.40E-06 | -0.8272 | down |
