# Supplementary figures and images for: A whole transcriptome profiling analysis for antidepressant mechanism of Xiaoyaosan mediated synapse loss via BDNF/trkB/PI3K signal axis in CUMS rats
Source: BMC Complement Med Ther. 2023 Jun 15;23:198. doi: 10.1186/s12906-023-04000-0 (PMC10273699; doi:10.1186/s12906-023-04000-0)

## AKT-56kDa

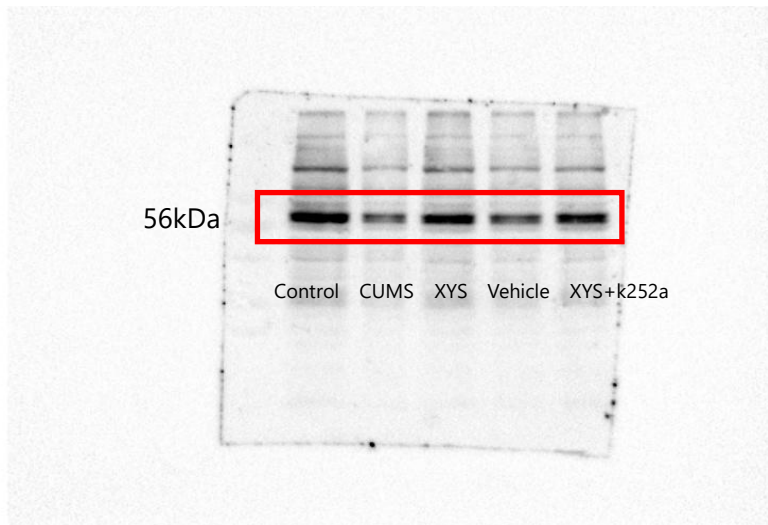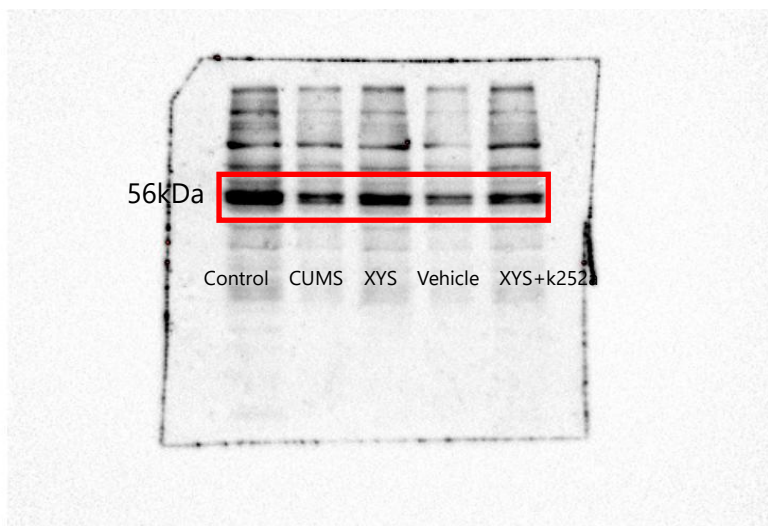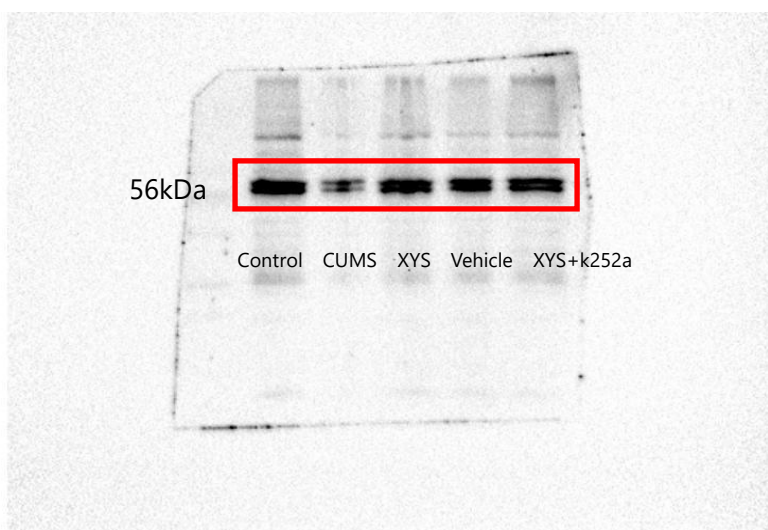

## PI3K-126kDa

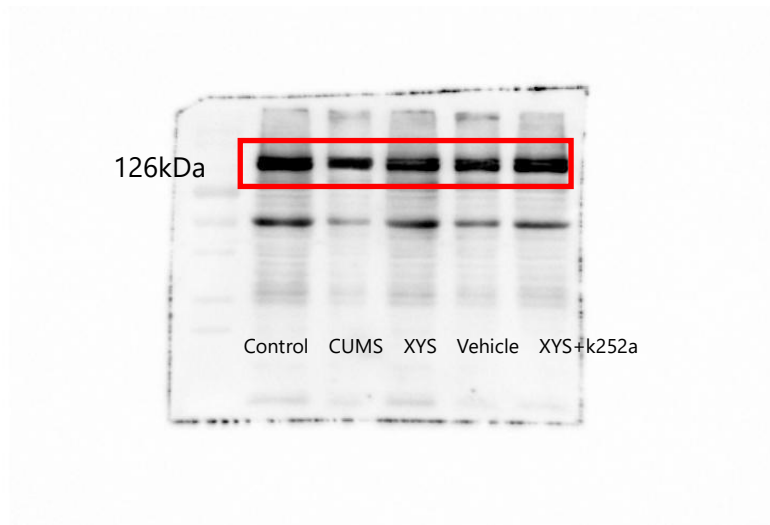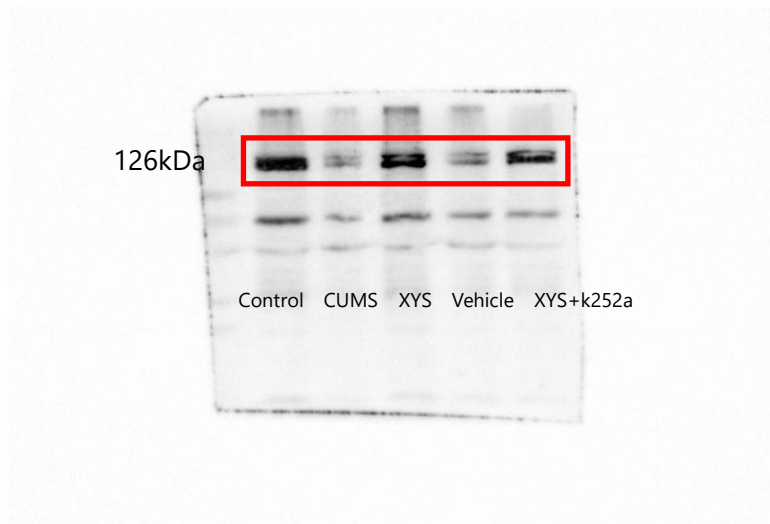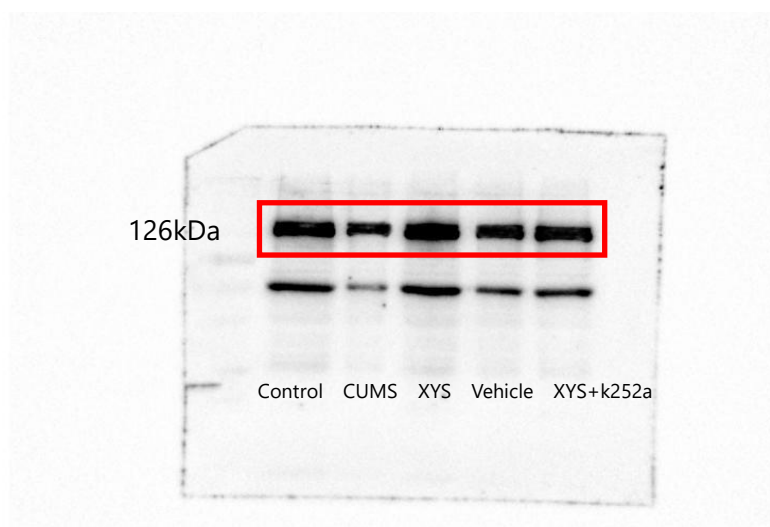

## TrKB-92kDa

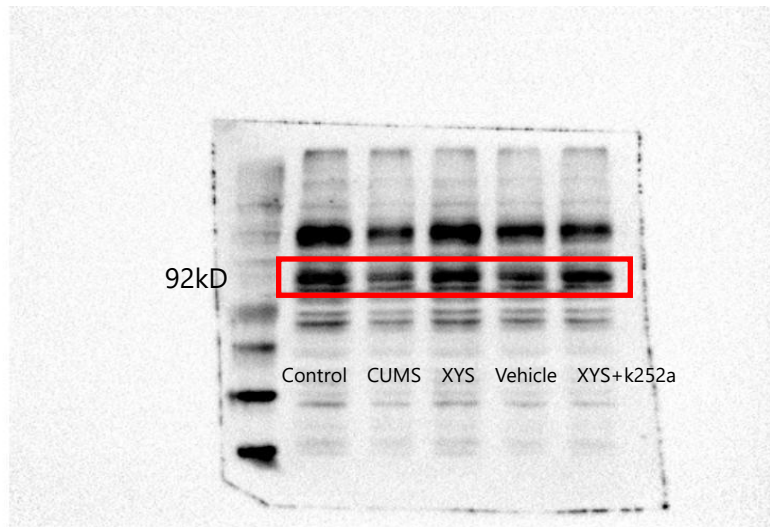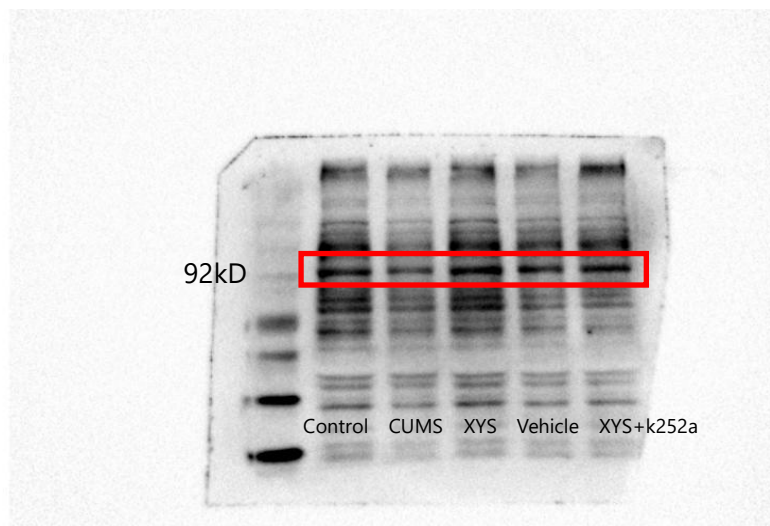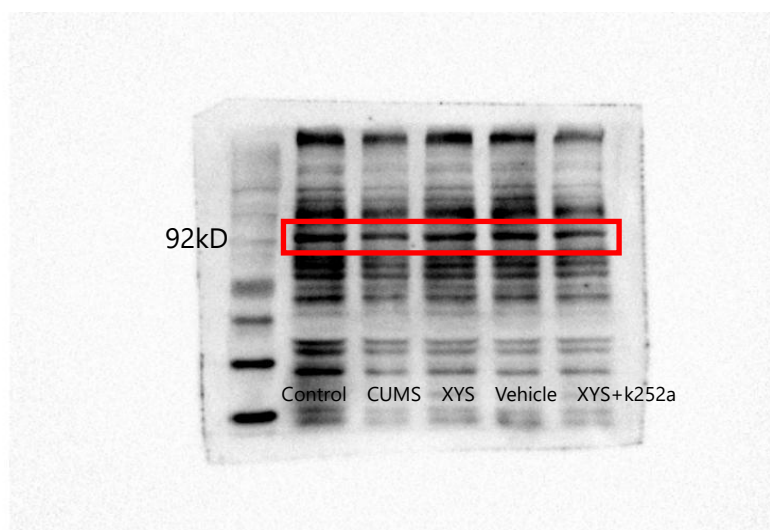

p-TrKB-145kDa

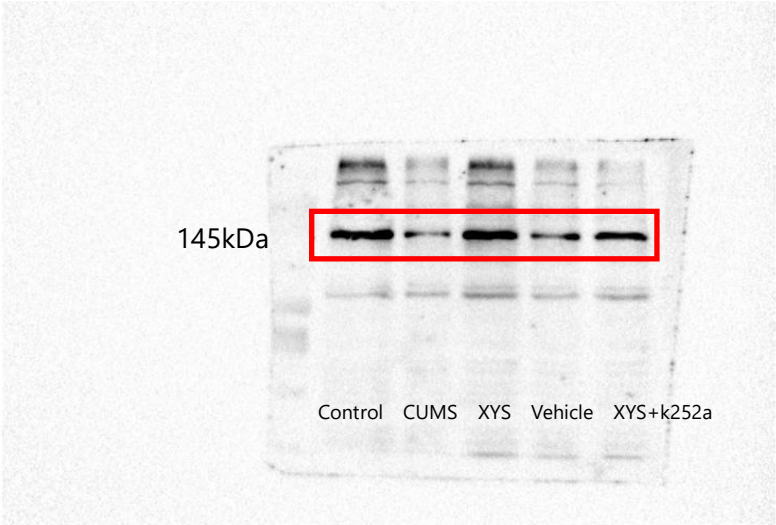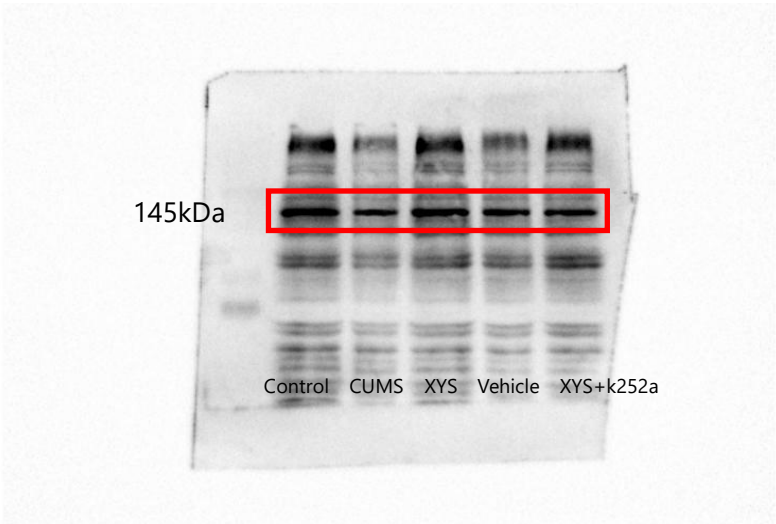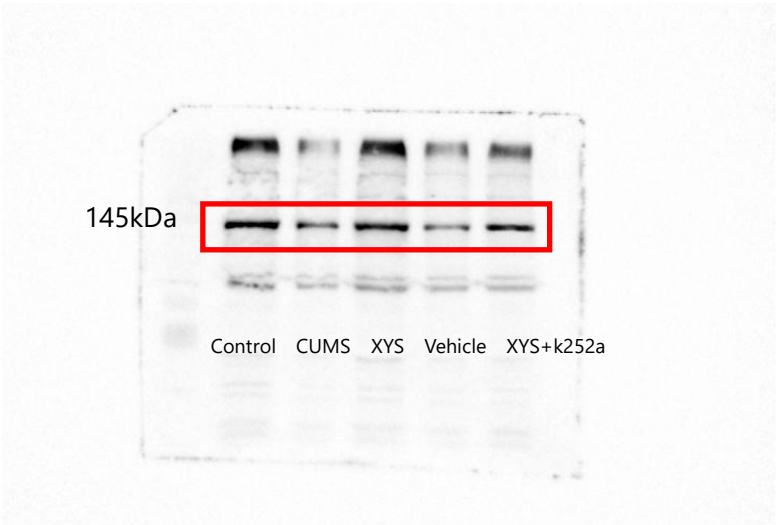

## p-AKT-62kDa

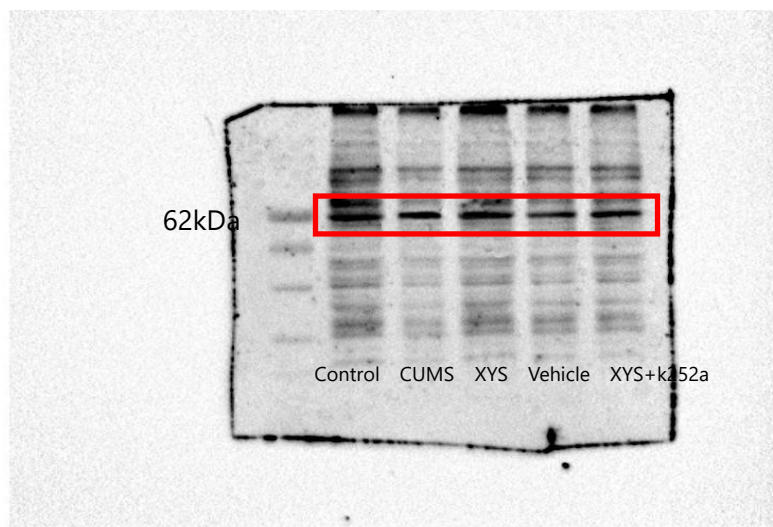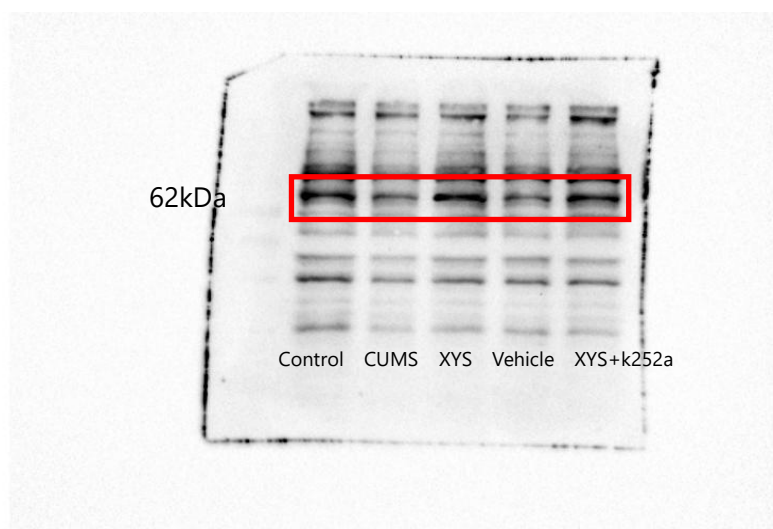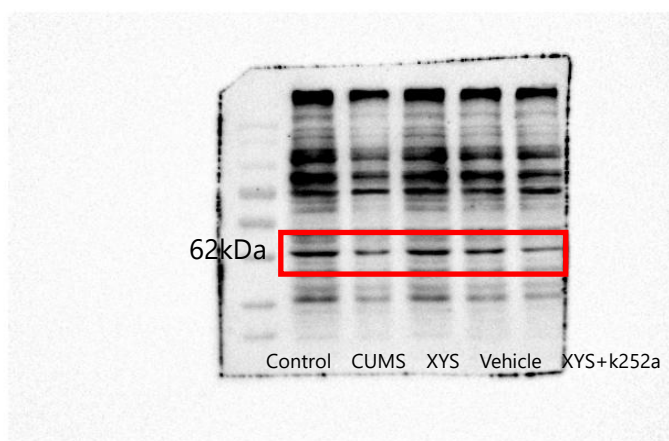

## BDNF-28kDa

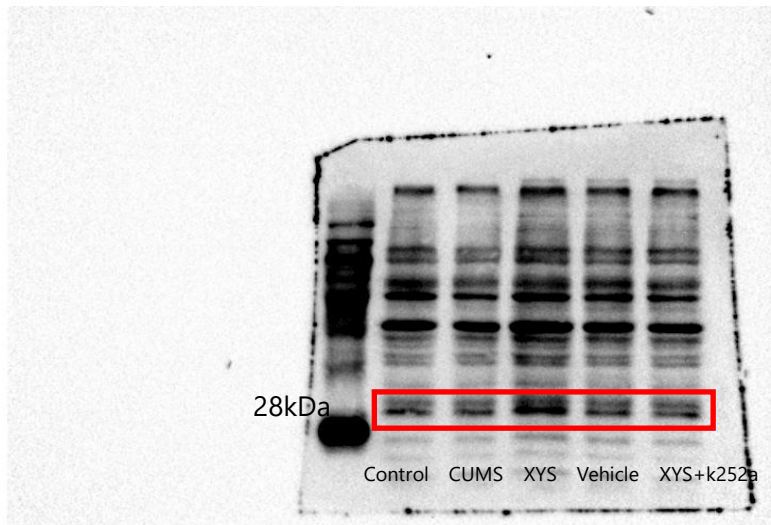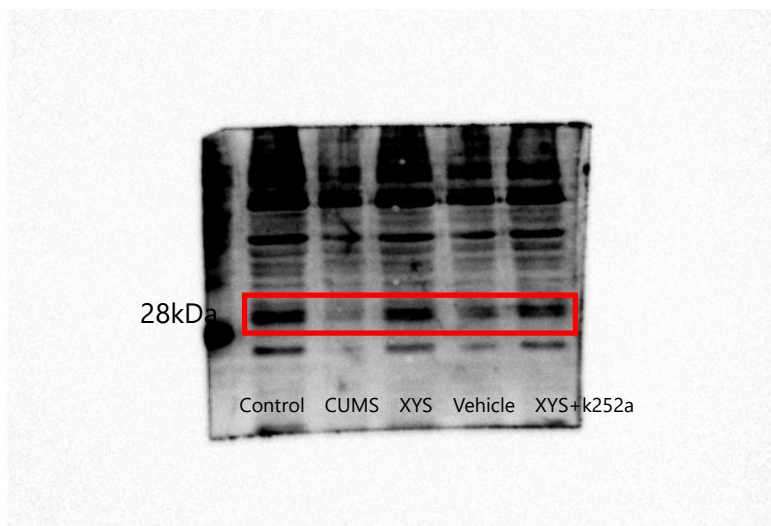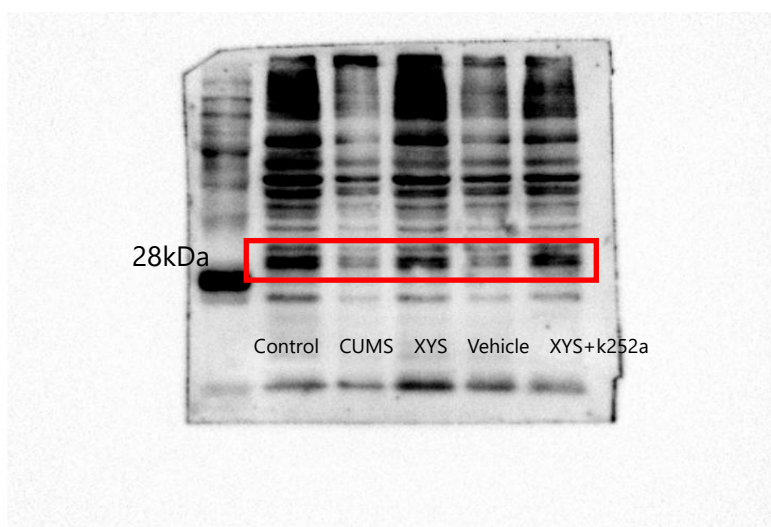

## $\beta$ -actin-42kDa

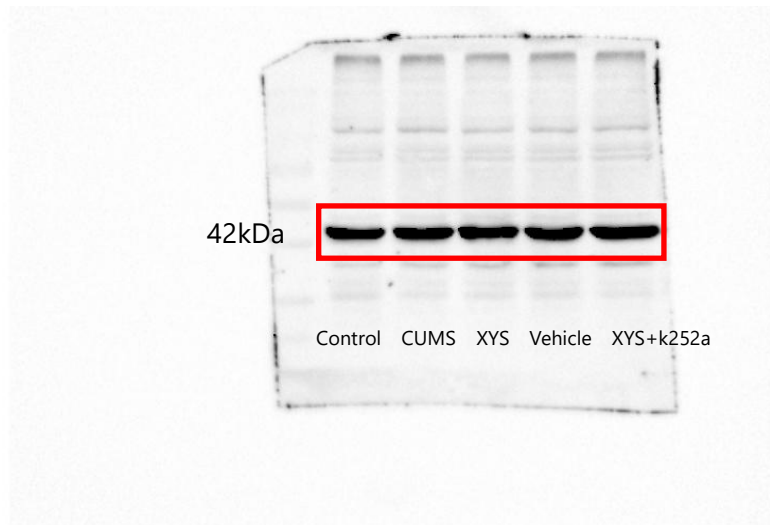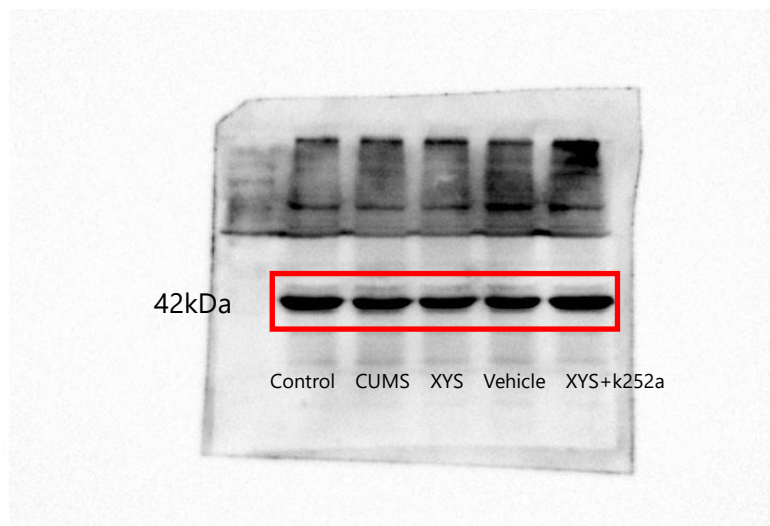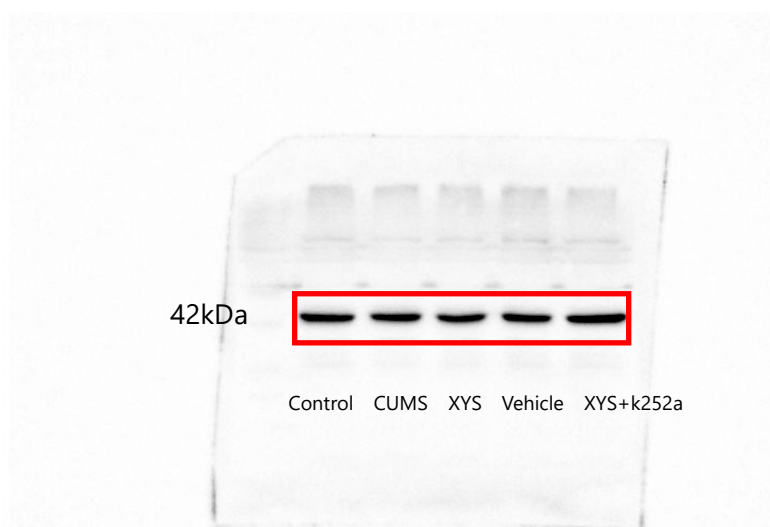

Supplement: Supplementary file 2 — Additional file 2. [file 12906_2023_4000_MOESM2_ESM.zip › WBú¿Gelú¬--3.8.pdf]
